# Distinct GSDMB protein isoforms and protease cleavage processes differentially control pyroptotic cell death and mitochondrial damage in cancer cells

**DOI:** 10.1038/s41418-023-01143-y

**Published:** 2023-03-11

**Authors:** Sara S. Oltra, Sara Colomo, Laura Sin, María Pérez-López, Sara Lázaro, Angela Molina-Crespo, Kyoung-Han Choi, David Ros-Pardo, Lidia Martínez, Saleta Morales, Cristina González-Paramos, Alba Orantes, Mario Soriano, Alberto Hernández, Ana Lluch, Federico Rojo, Joan Albanell, Paulino Gómez-Puertas, Jae-Kyun Ko, David Sarrió, Gema Moreno-Bueno

**Affiliations:** 1 Fundación MD Anderson Internacional, Madrid, Spain; 2 Departamento de Bioquímica, Universidad Autónoma de Madrid (UAM), Instituto de Investigaciones Biomédicas ‘Alberto Sols’, Conexión Cáncer (UAM-CSIC), IdiPAZ, Madrid, Spain; 3 Centro de Investigación Biomédica en Red de Cáncer (CIBERONC), Instituto de Salud Carlos III, Madrid, Spain; 4 Department of Surgery, The Ohio State University Wexner Medical Center Columbus, Columbus, OH 43210 USA; 5 Molecular Modeling Group, Centro de Biología Molecular Severo Ochoa, CBMSO Conexión Cáncer, (CSIC-UAM), Madrid, Spain; 6 Centro Nacional de Biotecnología (CNB), UAM-CSIC Madrid, Spain; 7 Servicio de Microscopía Electrónica, Centro de Investigación Príncipe Felipe (CIPF), Valencia, Spain; 8 Servicio de Microscopía Óptica Avanzada, Centro de Investigación Príncipe Felipe (CIPF), Valencia, Spain; 9 GEICAM, Spanish Breast Cancer Group, Madrid, Spain; 10 Hospital Clínico Universitario de Valencia, Valencia, Spain; 11 Instituto de Investigación Sanitaria INCLIVA, Universidad de Valencia, Valencia, Spain; 12 Hospital Universitario Fundación Jiménez Díaz, Madrid, Spain; 13 IMIM Hospital del Mar Medical Research Institute, Barcelona, Spain; 14 Medical Oncology Department Hospital del Mar, Barcelona, Barcelona, Spain; 15 Institut de Recerca Biomedica Barcelona, Barcelona, Spain

**Keywords:** Tumour biomarkers, Cancer

## Abstract

Gasdermin (GSDM)-mediated pyroptosis is functionally involved in multiple diseases, but Gasdermin-B (GSDMB) exhibit cell death-dependent and independent activities in several pathologies including cancer. When the GSDMB pore-forming N-terminal domain is released by Granzyme-A cleavage, it provokes cancer cell death, but uncleaved GSDMB promotes multiple pro-tumoral effects (invasion, metastasis, and drug resistance). To uncover the mechanisms of GSDMB pyroptosis, here we determined the GSDMB regions essential for cell death and described for the first time a differential role of the four translated GSDMB isoforms (*GSDMB1-4*, that differ in the alternative usage of exons 6-7) in this process. Accordingly, we here prove that exon 6 translation is essential for GSDMB mediated pyroptosis, and therefore, *GSDMB* isoforms lacking this exon (*GSDMB1-2*) cannot provoke cancer cell death. Consistently, in breast carcinomas the expression of *GSDMB2*, and not exon 6-containing variants (*GSDMB3-4*), associates with unfavourable clinical-pathological parameters. Mechanistically, we show that GSDMB N-terminal constructs containing exon-6 provoke cell membrane lysis and a concomitant mitochondrial damage. Moreover, we have identified specific residues within exon 6 and other regions of the N-terminal domain that are important for GSDMB-triggered cell death as well as for mitochondrial impairment. Additionally, we demonstrated that GSDMB cleavage by specific proteases (Granzyme-A, Neutrophil Elastase and caspases) have different effects on pyroptosis regulation. Thus, immunocyte-derived Granzyme-A can cleave all GSDMB isoforms, but in only those containing exon 6, this processing results in pyroptosis induction. By contrast, the cleavage of GSDMB isoforms by Neutrophil Elastase or caspases produces short N-terminal fragments with no cytotoxic activity, thus suggesting that these proteases act as inhibitory mechanisms of pyroptosis. Summarizing, our results have important implications for understanding the complex roles of GSDMB isoforms in cancer or other pathologies and for the future design of GSDMB-targeted therapies.

## Introduction

Pyroptosis is an inflammatory form of programmed cell death that involves cell swelling and lysis, which causes massive release of cellular contents and thereby triggers strong inflammation [1, 2]. Pyroptosis, which is activated in response to diverse damaging signals, has been involved in multiple pathologies, including infectious, inflammatory diseases and cancer [3–5]. Pyroptotic cell death is also called “Gasdermin-mediated programmed necrosis” [6, 7], since the Gasdermin (GSDM) family of pore-forming proteins plays a key role in this process. Except PJVK (Pejvakin), all GSDMs members (GSDMA/B/C/D/E) share similar protein 3D structure and a common pro-cell death activation mechanism [8–11]. Briefly, the N-terminal (NT) pore-forming domain is auto-inhibited by the C-terminal (CT) domain, and GSDM proteins remain inactive in the cytosol. After specific stimuli, the released NT binds to diverse lipids and forms pores in the cell membrane, as well as in mitochondria and/or other organelles, subsequently leading to cell death [4, 12–15]. For each GSDM, the active NT is released, in a biological context-dependent manner, by the cleavage of the linker inter-domain region via specific cell-intrinsic or extrinsic proteases. For instance, GSDMA-NT is cleaved by Streptococcal pyrogenic exotoxin B [16], GSDMB through lymphocyte-derived Granzyme A (GZMA) [17, 18], GSDMC by caspase 6/8, GSDMD by pro-inflammatory caspases (caspases1/4/5/11) [19–22], apoptotic Caspase-8 [23], Neutrophil Elastase (NE/ELANE) [24], or Cathepsin G [25], and GSDME via Caspase-3 or GZMB [18, 26]. Moreover, alternative cleavage events can inactivate the NT pore-forming capacity of some GSDMs [10, 27–29].

GSDMB plays complex biological roles since it can exhibit either cell-death dependent and independent functions in diverse pathological conditions such as enterobacteria infection [30], asthma [31, 32], inflammatory bowel diseases [29, 33–35], and cancer [15, 36, 37]. *GSDMB* is expressed in diverse organs (mostly in gastrointestinal tract, respiratory system, lymphoid tissues [38]) and in multiple tumor types (including breast, gastric and bladder, among others) [37]. In tumors, *GSDMB* promotes either pro-tumor or anti-tumor functions depending on the biological context [37]. *GSDMB* is frequently co-expressed with *HER2/Erbb2* oncogene in breast and gastric carcinomas [39, 40], where *GSDMB* over-expression promotes tumorigenesis [41], invasion, metastasis, and resistance to therapy [39, 42]. Paradoxically, GSDMB can also have an anti-tumour role if its pore-forming pyroptotic function is activated in cancer cells. This activation can be trigger either with a GSDMB-targeted nanotherapy [42] or extrinsically through its cleavage by GZMA [17]. In a context of immune activation, NK and T-cells secrete GZMA, which in turn cleaves GSDMB either at the K229 or K244 residues within cancer cells, inducing pyroptosis and the subsequent antitumoral immune response [17]. Therefore, triggering GSDMB pyroptotis has been proposed as a promising approach for efficient tumor killing. However, to develop future GSDMB-targeted treatment approaches it is essential first to fully define the precise functional domains and the regulatory mechanisms of GSDMB pyroptotic activity, since there are controversial and contradictory results. Aside GZMA cleavage [17], Panganiban and collaborators [37] proposed that caspase 1 cleaved GSDMB (after D236 in the linker region) releasing a pyroptotic NT fragment, while Chao et al. [10] and Chen et al. [29] showed that many caspases could cleave GSDMB within the NT domain (D91) producing a cell-death inactive protein. Moreover, Shi et al. [43] reported that GSDMB NT domain did not induce cell death. Finally, Ding et al. [8] generated a GSDMB construct of 275 aminoacids (including part of the CT region) that produced strong lytic death, though it is unknown if any protease could generate this fragment.

Of note, it is important to highlight that there are different GSDMB protein variants but their potentially distinct functional relevance in physiology and disease is usually overlooked. Indeed, *GSDMB* gene produces at least six transcripts (NCBI Gene ID: 55876) that are translated into four different protein variants (hereafter termed GSDMB 1-4). The tissue-specific expression patterns (GTEx Portal) [44] of these variants are controlled by genetic features like SNPs [32, 45] and other complex regulatory mechanisms [15, 46, 47]. The four translated isoforms differ only in the presence of exon 6 (13 aminoacids, aa) and exon 7 (9 aa): *GSDMB transcript variant 1* (*GSDMB1*) lacks exon 6 (Δ6), *GSDMB2* lacks exons 6 and 7 (Δ6-7), *GSDMB3* contains both, and *GSDMB4* lacks exon 7 (Δ7). The residues translated by these exons are located within the flexible inter-domain linker region of the protein [10], but their biological function remained unclear so far. While recent reports indicate that these variants could mediate different effects both in cancer [48] and inflammatory diseases [31], their precise regulatory mechanisms and their involvement in pyroptosis and other GSDMB functions is largely unknown.

To shed light into the mechanism of GSDMB cell death induction herein we determined the GSDMB regions essential for pyroptosis and subsequently we described a differential role of the distinct GSDMB isoforms in this process. Moreover, our data indicate that GSDMB-driven cell death is associated with a concomitant mitochondrial damage. Furthermore, we have also demonstrated that specific proteases can generate different pyroptotic active or inactive GSDMB fragments. Importantly our data also revealed that the expression of *GSDMB* pyroptotic-proficient and -deficient isoforms differentially correlate with clinic-pathological parameters in breast carcinomas. This pioneering study clarifies the mechanisms of GSDMB pyroptosis and highlight the distinct relevance of GSDMB isoforms in cancer biology.

## Materials/Subjects and methods

Expanded and detailed information of the methods is provided in Supplementary Information.

### Human samples and ethics

Breast cancer samples are part of a multicenter, prospective, observational study [49] coordinated by GEICAM (Spanish Group for Breast Cancer Research) with the participation of 31 Spanish hospitals. The study protocol was approved by the Institutional Review Board and the Ethics Committee of Hospital Provincial de Castellón (Spain), according to the requirements of the Spanish regulations (GEICAM 2009-03; clinicaltrials.gov identifier: NCT01377363). Written informed consent was obtained from all patients before enrollment. In these tumors quantitative expression of GSDMB isoforms were performed (see Supplementary Materials and Methods).

### Cell biology methods

HEK293T, SKBR3, 23132/87 and THP1 cell lines were grown according to the standard conditions (Supplementary Table 1). Transient transfection of all constructs and the corresponding empty vectors (Supplementary Materials and Methods) was performed using lipofectamine 2000 (Invitrogen) according to the manufacturers’ protocol. To evaluate GSDMB cytotoxicity and mitochondrial damage, Lactate Dehydrogenase (LDH) release tests (Roche), MitoSOX (Thermo Fisher), TMRE (abcam) and flow cytometry Caspase 3/7-SYTOX (Thermo Fisher) assays were performed according to manufacturers’ instructions. Mitochondrial DNA release was performed essentially as described before [50].

To assess proteolysis induced by Neutrophil Elastase (NE), cell lysates from transfected HEK293T cells were incubated at 37 °C for 1 h with different rhNE (Sigma) concentrations. When indicated, BAY-678 inhibitor was included. GSDMB cleavage bands were evaluated by In-Gel Digestion and reverse phase-liquid chromatography RP-LC-MS/MS analysis. Co-culture of HEK293T or SKBR3 cells with NK-92 cell line was performed as described before [17]. The cleavage products were visualized by Western blot (WB). Uncropped original WBs are included as Supplementary Information. All antibodies used in the study are included in Supplementary Table 2.

### Microscopy methods

GSDMB constructs were visualized by immunofluorescence in transient transfected (72 h) and 4% paraformaldehyde-fixed cells with the indicated primary antibodies (Supplementary Table 2) as described before [42]. Confocal microscopy images were captured by LSM710 microscope (Zeiss) and processed by Fiji software (Image J 1.52). Alternatively, live cell tracking imaging was performed with doxycycline inducible vectors expressing GFP-tagged GSDMB constructs (Supplementary information). To evaluate mitochondrial morphology correlative light and electron microscopy (CLEM) procedures were performed in 23132/87 cells. Cells were seeded in a permanox Lab-Tek chamber slide (Nalge Nunc International) and transfected with doxycycline inducible vectors. After 6 h of transfection, cells were induced with Doxycycline at 200 ng/ml, incubated with red MitoTracker^TM^ Deep Red FM (ThermoFisher) for 30 min at 37 °C and fixed in 3% glutaraldehyde. The confocal images were acquired with a Leica TCS SP8 HyVolution II (Leica Microsystems). After fluorescence capture, slides were processed for transmission electron microscopy analysis with FEI Tecnai Spirit BioTwin (ThermoFisher). Pictures were taken using Radius software (Version 2.1) with a Xarosa digital camera (EMSIS GmbH).

### The Cancer Genome Atlas (TCGA) data analysis

GSDMB transcript variants mRNA expression from breast cancer patients was analysed using mean-normalized RNAseq data from TCGA [51]. Expression data from 1093 breast cancer patients with follow up information were analysed. Briefly, *GSDMB1-4* mean expression values were used as cut-off to classify tumours with high (> mean) and low (≤ mean) GSDMB variants expression. Then, the correlation of *GSDMB* isoform expression and overall survival was obtained using “survival” R package (R Bioconductor). P-values were calculated using log-rank test. Hazard ratio was calculated by Cox proportional-hazards model. Results were considered significant when *p*-value < 0.05.

### Statistical analysis

Analysis of statistical significance for the indicated datasets was performed with ANOVA or unpaired t-test using GraphPad Prism 8 (GraphPad Software). The statistical test used for each assay is detailed in the Figure legends. All experiments were repeated at least three times, and the number of experimental replicates is specified in the Figure Legends. Results were considered statistically significant when *p*-value < 0.05.

## Results

### Only the GSDMB isoforms containing exon 6 present cytotoxic activity

**Fig. 1:**
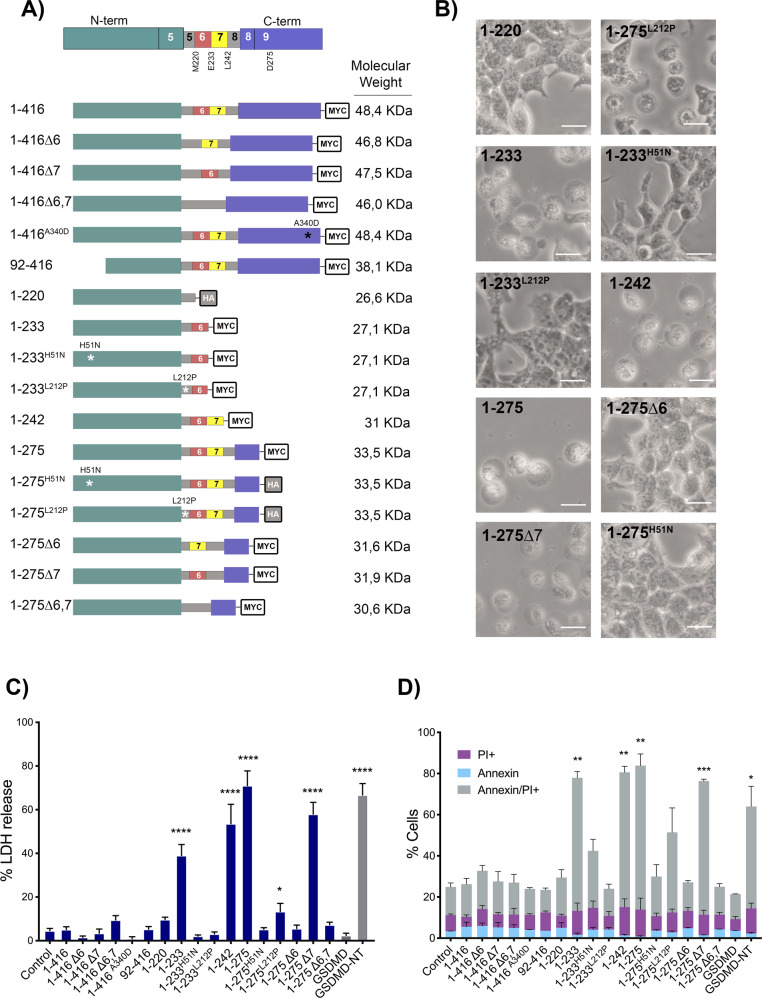
GSDMB exon 6 is essential for GSDMB-NT mediated pyroptosis. **A** Top: Scheme of full length GSDMB protein, indicating the NT domain (green), the CT domain (purple). The figure also indicates the approximate location of the last residue encoded by exons 5 (M220), exon 6 (E233) and exon 7 (L242) as well as the D275 residue (located on exon 9, end of the 1–275 cytotoxic construct reported by Ding et al 2016. The flexible linker interdomain region (gray) comprises aminoacids Q210 to S252 and can contain the residues encoded by the alternative exons 6 (red) and 7 (yellow). Below: schematic representation of the different GSDMB constructs used, indicating the localization of truncations or point mutations, and C-terminal tags (myc or HA). GSDMB translated variants GSDMB-1 to 4 are named 1–416Δ6, 1–416Δ6,7, 1–416, 1–416Δ7, respectively. **B** Representative bright field microscopy images of cells transiently transfected (48 h) with the indicated GSDMB constructs. Scale bar represents 20 μm**. C** Cytotoxicity of GSDMB constructs measured by lactate dehydrogenase (LDH) release assay in transiently transfected (48 h) HEK293T cells. Empty vector (Control) and full-length GSDMD were used as a negative controls and GSDMD-NT as a positive control. Values indicate the percentage of LDH release and represent means ± SEMs of six independent experiments. Differences between control condition (empty vector) and each GSDMB construct were tested by two-tailed unpaired t-test: *p < 0,05 and ****p < 0,0001. **D** Annexin V/PI cell death analyzed by flow cytometry. Values represents the percentage of cells positive for PI (purple), annexin V (light blue) and doble Annexin V/PI (grey). Values represent means ± SEMs of 3 independent experiments. Statistical analyses were performed by two-tailed unpaired t-test, comparing Annexin/PI positive cells from each condition vs. empty vector control. *p < 0,05, **p < 0,01 and ***p < 0,001.

To assess the functional importance on cell death induction of different regions of GSDMB protein including the alternative exons 6 and 7, multiple myc/HA-tagged GSDMB constructs were used (Fig. 1A). These constructs were named following the exon codifying number and the aminoacid sequence of the longest translated GSDMB variant (GSDMB3, 416 aminoacids). Hence, the four translated isoforms, differing in the presence of exons 6 and 7, were termed 1–416Δ6 (GSDMB1), 1–416Δ6,7 (GSDMB2), 1–416 (GSDMB3) and 1–416Δ7 (GSDMB4). We also cloned different NT constructs ending after the translation of either exon 5 (1–220; shared by all isoforms), exon 6 (1–233), exon 7 (1–242), as well as the 1–275 fragment (containing the linker and part of the CT region encoded by exon 9), previously described as pyroptotic [8], and its respective isoform variants (lacking exons 6 and/or 7). Moreover, during the cloning process of 1–275, two spontaneous mutations were originated, and these constructs (1–275^H51N^ and 1–275^L212P^) were also included in the study. We later generated the same point mutations within the 1–233 fragment (1–233^H51N^ and 1–233^L212P^). Additionally, within 1–416 we introduced the A340D point mutation, an amino acid change that release the NT-CT autoinhibition and induces pyroptosis in other GSDMs [8]. Finally, we cloned the CT fragment (92–416) that occurs when caspase-3 cleaves (after D91) in the GSDMB-NT region [10].

To evaluate the pyroptotic potential of these constructs, they were transiently transfected (48 h, >60% transfection rates; Supplementary Fig. 1A–C) in HEK293T cells, which do not express endogenous *GSDMs* (Supplementary Fig. 1B) [17]. Like the GSDMD-NT (1–275) pyroptotic domain [8], used as positive control, only those cells transfected with 1–233, 1–242, 1–275, 1–275^L212P^ or 1–275Δ7 GSDMB constructs exhibited pyroptotic morphology with remarkable cell swelling (Fig. 1B), and produced a significant increase in LDH release and cell death rates by annexin/IP assays (Fig. 1C, D). Flow cytometry analyses of Caspase3/7 activation and Sytox Green cell dye confirmed that GSDMB-NT cytotoxic fragments induced pyroptosis but not apoptosis (Supplementary Fig. 2A).

These initial results provide novel insights into GSDMB pyroptotic function: a) Under unstimulated conditions, neither the full-length (not cleaved) GSDMB transcripts variants nor the 92–416 construct (described before [10]) produce lytic cell death; b) Among the NT constructs tested, the 1–233 (including exon 6) is the minimum fragment with pyroptotic activity, as 1–220 (till exon 5) is not cytotoxic; c) The translation of alternative exon 6, but not exon 7, is required for pyroptosis, since all cytotoxic constructs (1–233, 1–242, 1–275, 1–275^L212P^ and 1–275Δ7) contains exon 6. In fact, removal of exon 6 but not exon 7, blocks cell death (compare 1–275 with 1–275Δ6, 1–275Δ7 or 1–275Δ6,7). This implies that only GSDMB isoforms 3 and 4 and not 1 or 2 (which lack exon 6) could have activatable pyroptotic activity; d) For GSDMB-NT cytotoxicity, the Histidine 51 residue is essential (H51N mutation completely blocks cell lysis in 1–233 and 1–275 fragments), while Leucine-212, has moderate relevance (L212P mutation inhibits 1–233 cytotoxicity but only partially reduces 1–275 cell death promotion; Fig. 1C, Supplementary Table 3). Multiple sequence alignment of all GSDMs revealed that the L212 residue, but not the H51, is highly conserved among GSDM family members (Supplementary Fig. 2B). e) Finally, contrary to other GSDMs members [8], the mutation of a conserved Alanine in the CT region (A340) does not release autoinhibition and activate pyroptosis of GSDMB.

**Fig. 2:**
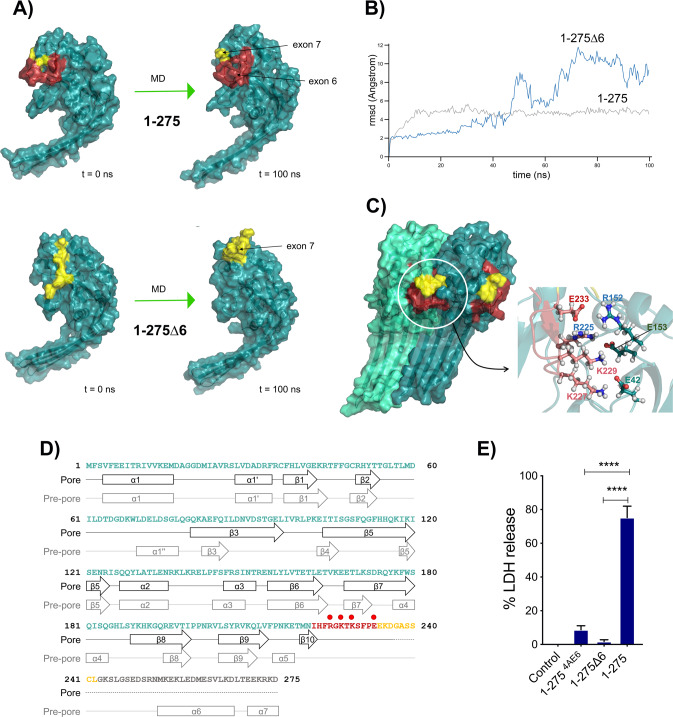
Structural modeling of 1–275 cytotoxic fragment and molecular dynamics (MD) simulation studies reveal a key function of exon 6 residues in GSDMB pyroptosis. **A** Structural model of the N-terminal fragments of 1–275 (top) and 1–275Δ6 (without exon 6, bottom) before (left) and after (right) 100 ns of unrestricted molecular dynamics simulation. The position of the amino acids corresponding to exon 6 (red) and exon 7 (yellow), all located on the homo-dimerization surface of the structure, is indicated. **B** Root-mean square deviation (rmsd, Angstrom) values corresponding to the amino acids of exons 6 and 7 (1–275, grey line) or exon 7 (1–275Δ6, blue line) during the 100 ns of MD simulation procedure. **C** Model structure of a dimer of the 1–275 NT fragment after 100 ns of unrestricted molecular dynamics. Amino acids belonging to exons 6 and 7 are colored red and yellow, respectively. The figure on the left shows how the amino acids of exon 6 form part of the interaction surface between the monomers. The inset on the right shows in detail the position of the charged amino acids R225, K227, K229 and E233, of exon 6, close to the position of the complementary charged amino acids E42, R152 and E153, located in the surface of the adjacent monomer. **D** Secondary structure elements predicted for the model of the 1–275 NT fragment, both in the pre-cleavage form (Pre-pore) and in the post-cleavage form that is part of the polymeric structure of the transmembrane pore (Pore). Residues belonging to exons 6 and 7 are colored red and yellow, respectively. The position of residues R225, K227, K229 and E233 of exon 6 is indicated (red dots). **E** Cytotoxicity of the 1–275^4AE6^ construct, in which four amino acids located in the contact monomer position have been mutated to Alanine (R225A, K227A, K229A, E233A), was measured by LDH release and compared with the wild type 1–275. Values represent means ± SEMs of 3 independent experiments, statistical analyses were performed by two-tailed unpaired t-test, comparing the cytotoxic N-terminal 1–275 with the rest of N-terminal constructs: ****p < 0,0001.

To ascertain the relevance of exon 6 amino acids on the GSDMB structure and its pore-forming ability, we generated a 3D model of the 1–275 NT with and without exon 6 (See Supplementary Materials and Methods) and these structures were subsequently subjected to 100 ns of unconstrained molecular dynamics (MD) simulation. According to the models, exons 6 and 7 residues are located on the homo-dimerization surface of GSDMB NT fragment (Fig. 2A). However, the deletion of exon 6 (1–275Δ6) during the MD simulation provokes a noticeable modification in the position of exon 7 (Fig. 2A, B-blue line-), compared to the 1–275 model containing both exons (Fig. 2A, B-grey line-). As exon 6 and 7 amino acids are located on the homo-dimerization surface implicated in the formation of the membrane pore, the simulation suggests that the absence of exon 6 compromises the correct homo-dimerization and the formation of the pore in the 1–275 cytotoxic fragment.

Moreover, we investigated if exon 6 residues were involved in the monomer-monomer interaction during the formation of the transmembrane pore by generating a model of NT GSDMB dimer (Fig. 2C), using data from the polymeric structures of GSDMA3 [52] and human GSDMD [53] NTs. After MD simulation, (Fig. 2C, D) we identified a set of charged amino acids in the exon 6 (R225, K227, K229 and E233) that locate close to a group of amino acids with complementary charges (E42, R152, E153) present on the adjacent monomer surface. Some of these residues, conserved in other GSDMs, have been involved in oligomerization and lipid binding of GSDMD [9, 52, 53]. Thus, to verify the role of these residues in GSDMB pyroptosis, we mutated these four residues to Alanine (R225A, K227A, K229A, E233 A) in the 1–275 fragment (called 1–275^4AE6^) and analyzed its effect on LDH release in HEK293T cells. Alike exon 6 complete deletion (1–275Δ6), the combined mutation of these amino acids significantly reduces LDH release compared to 1–275 (Fig. 2E).

Collectively, our data reveal that the amino acids of exon 6 are directly involved in the stability of the polymeric structure forming the transmembrane pore and are, thus, implicated in the pyroptotic induction.

### GSDMB NT-induced cell death associates with mitochondrial damage

**Fig. 3:**
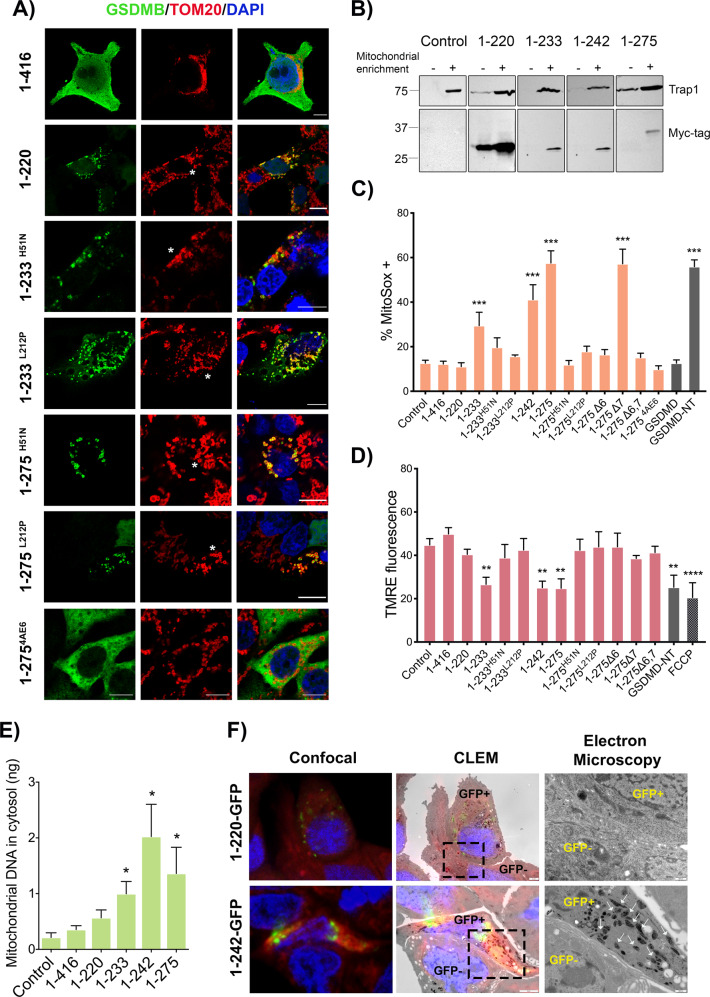
Mitochondrial localization of cytotoxic GSDMB-NT constructs associates with mitochondrial damage. HEK293T cells were transiently transfected (48 h) with the indicated GSDMB constructs. **A** Intracellular localization of GSDMB constructs (green; NT antibody SIGMA) and co-localization with mitochondria (red; TOM20) by immunofluorescence and confocal microscopy analysis. Note that GSDMB-NT constructs mostly localize in dot-like or round-shaped mitochondria (asterisks). Nuclei was stained with DAPI (blue). Scale bar represents 10 μm. **B** Mitochondrial localization of cytotoxic GSDMB-NT constructs by immunoblot in cytosolic and mitochondrial enriched fractions. Cytosol (C) and mitochondrial (M) fractions were analyzed by immunoblot probed for Myc-tag and Trap1. Mitochondrial damage was analyzed in transiently transfected HEK293T cells with mitoSOX assay using flow cytometry (48 h post-transfection) (**C**) or with fluorescence assay to measure membrane potential (TMRE) (**D**). **E** Quantification of mitochondrial DNA (mtDNA) presence in cytosolic fraction of HEK293T cells was analyzed by qRT-PCR. The 1-416 control was used as negative control because it does colocalize with mitochondria. MitoSOX, TMRE or mtDNA release values represent means ± SEMs of eight independent experiments. Differences between empty vector control and each GSDMB construct were tested by two-tailed unpaired t-test: *p < 0,05, **p < 0,01 and ***p < 0,001. GSDMD-NT was used as a positive control and, in TMRE assay, FCCP (carbonilcyanide p-triflouromethoxyphenylhydrazone) was used as a positive control for reducing mitochondrial membrane potential. **F** Correlative light-electron microscopy (CLEM) study in 23132/87 cell lines. The expression of 1–220-GFP or 1–242-GFP GSDMB-NT constructs was induced with Doxycycline. Mitochondria was labelled by mitoTracker Deep Red. Cells were analyzed using CLEM technique that combines immunofluorescence confocal and electron microscopy. The first column depicts confocal microscopy images showing GSDMB-GFP (green) and mitochondria (red). The second column shows the same area with the overlay confocal/electron microscope (CLEM). The last column shows a magnified image (by electron microscopy) of the dotted area indicated in the CLEM picture. Cells expressing the indicated GSDMB-NT-GFP construct are labelled as GFP + and adjacent negative cells are indicated as GFP-. White arrows indicate high electrodense mitochondria in 1–242-GFP positive cells.

Next, to assess the intracellular localization of our GSDMB constructs we performed immunofluorescence and confocal microscopy analysis using transiently transfected HEK293T cells. The constructs with point mutations that reduce the pyroptotic effect (1–233^H51N^, 1–233^L212P^, 1–275^H51N^ and 1–275^L212P^) and the 1–220 NT fragment mostly accumulate as dot-like or ring-shape aggregates (Fig. 3A and Supplementary Fig. 3A, B). These aggregates mainly co-localized with the specific mitochondrial markers TOM20 (Fig. 3A), HSP75/Trap-1 and mitoTracker Deep Red (Supplementary Fig. 4), and not with lysosomes (LAMP1) or the Golgi apparatus (GM130) (Supplementary Fig. 5). The 1–275^4AE6^ construct shows a diffuse cytoplasmic localization without mitochondrial accumulation, similar to the results obtained when exon 6 is missing (1–275Δ6) (Fig. 3A). Despite we were unable to visualize the most cytotoxic NT forms (1–233, 1–242, 1–275 and 1–275Δ7) by immunofluorescence in fixed cells, as dead cells readily detached from the coverslip, we confirmed that these proteins could be detected in purified mitochondrial fractions by WB (Fig. 3B). The remaining non-cytotoxic constructs were diffusely localized in the cytosol and showed no evident enrichment in any of the organelles tested (Supplementary Figs. 4, 5). Interestingly, the accumulation of GSDMB-NT fragments mainly associated with small mitochondria or with annular morphology (Fig. 3A-asterisk-), suggesting a potential dysfunction of this organelle.

In agreement with our data, the NT domain of other GSDMs not only localize to the cell membrane [8, 22, 54, 55] but also target mitochondria [12, 14, 19], and likely other organelles [14]. Therefore, to assess if GSDMB cytotoxicity also associated with mitochondria dysfunction we measured mitochondrial ROS (mitoSOX reagent), loss of membrane potential (TMRE), and mitochondrial DNA (mtDNA) release, as reported before [12, 43, 56–60]. Our findings revealed an overall positive association between the cytotoxic capacity (LDH release) and the mitochondrial dysfunction in the constructs analyzed (Supplementary Table 3). Thus, the cytotoxic 1–233, 1–242 and 1–275 fragments exhibited a sharp increase in mROS levels and mtDNA release, coupled with a decrease of mitochondrial membrane potential (Fig. 3C–E, Supplementary Table 3) Conversely, the mutations that reduce pyroptosis (1–233^H51N^, 1–233^L212P^, 1–275^H51N^, 1–275^4AE6^) and the 1–220 fragment produce no significant effect on these parameters, (Fig. 3C–E), despite these constructs mostly localized at the mitochondria (Fig. 3A).

To analyze if the dysfunction in the analyzed mitochondrial parameters translated into alterations at the mitochondrial ultra-structure, we used the correlative light-electron microscopy (CLEM) technique in 23132/87 cancer cells. For this, we generated GSDMB constructs fused (in the CT) with green fluorescence protein (GFP) using doxycycline-inducible lentiviral vectors (Supplementary Fig. 6). Both 1–220 or 1–242 GFP-tagged inducible constructs focally co-localized with mitoTracker Deep Red marker (Fig. 3F), but only cells expressing 1–242 (not 1–220 or the adjacent GFP-negative cells) exhibited highly electro-dense mitochondrial matrix, suggesting an effect of 1–242 NT on mitochondrial energy balance [61].

Together, these results indicate that GSDMB-NT induced cell death associates with mitochondrial disfunction. While highly cytotoxic fragments cannot be visualized by standard confocal imaging, point mutations inhibiting its killing activity allows its detection as aggregates into mitochondria. Despite abnormal appearance of mitochondria, these mutant constructs are incapable of producing measurable mitochondrial disfunction. Moreover, the data proves that exon 6 is required for GSDMB-NT mediated cell death and mitochondrial damage, but not for mitochondrial localization, since the 1-220 construct (until exon 5) show the strongest mitochondria accumulation (and not mitochondrial damage, Fig. 3A–F).

**Fig. 4:**
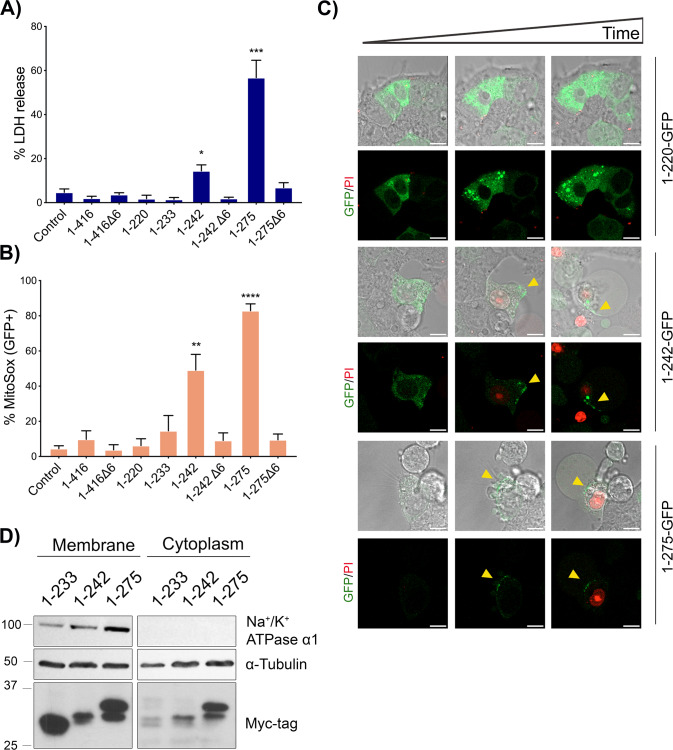
Cell membrane targeting of GSDMB-NT during pyroptosis. GSDMB-GFP constructs were transiently transfected (24 h) in HEK293T, and then the expression was induced by doxycycline. **A** Cytotoxicity was measured using lactate dehydrogenase (LDH) assay after 16 h of induction and (**B**) Mitochondrial damage of GFP positive cells was analyzed by mitoSOX using flow cytometry. Values represent means ± SEMs of five independent experiments. Differences between control condition and each GSDMB-GFP construct were tested by two-tailed unpaired t-test: *p < 0,05, **p < 0,01 and ***p < 0,001. **C** Microscopy imaging of GSDMB-NT localization in HEK293T during pyroptosis. Doxycycline inducible GSDMB 1–220-GFP, 1–242-GPF and 1–275-GFP were transiently transfected in HEK293T cells. Images are representative photograms (taken 1–2 h after doxycycline addition) from a time-lapse study. For complete videos see Supplementary Videos 1–12. Scale bar represents 10 μm. Green represents GSDMB-GFP constructs and red Propidium Iodide. **D** GSDMB-NT cytotoxic constructs localize in plasma membrane. Protein lysates from transfected HEK293T cells lysates were enriched using Plasma Membrane Protein Extraction Kit (Abcam). Cytoplasm and membrane fractions were analyzed by immunoblot probed for Myc-tag, α -tubulin and Na^+^/K^+^ ATPase α1.

In addition, to mitochondria targeting, our data prove that GSDMB-NT provoke cell lysis and release of intracellular LDH (Fig. 1), suggesting that pores are formed in the cell membrane. Since we were unable to detect membrane localization by standard confocal immunofluorescence in transiently transfected and fixed cells, to track GSDMB intracellular localization and to determine the pyroptosis kinetics as well as cell fate in real time, we performed time lapse fluorescence microscopy with multiple GFP-tagged doxycycline-inducible constructs Supplementary Fig. 6). After doxycycline induction, these constructs had the same effect that untagged ones in terms of cytotoxicity and mitochondrial ROS (Fig. 4A, B). The only exception was 1–233-GFP, which lost pyroptotic capability, likely due to GFP tagging, as reported before in a similar construct [19, 26]. Besides, the new construct 1–242Δ6-GFP did not exhibit pyroptosis, as expected. Live cell imaging of doxycycline-treated HEK293T cells reveals that (Fig. 4C, Supplementary Videos 1–12): a) initially all constructs show diffuse localization, but only the NT fragments (not full-length 1–416, Supplementary Videos 1–2) gradually form aggregates (even ring-shape structures, mostly seen in 1–220-GFP, 1–275Δ6-GFP and 1–275 Δ6; Supplementary Videos 3–8) that increased in size with time; b) the aggregates move throughout the cell body and occasionally localize to the cell membranes (Fig. 4C, Supplementary Videos 3–8); c) importantly, membranous localization of 1–242-GFP and 1–275-GFP (Fig. 4C, arrows) resulted in membrane lysis, cell swelling and massive cell death as revealed by Propidium Iodide (PI) uptake (Fig. 4C, Supplementary Videos 9–12) whereas augmented expression/aggregation or membrane localization of the other constructs did not provoke pyroptosis (Supplementary Videos 3–8). Besides, to confirm that, in addition to mitochondria (Fig. 3B), 1–233, 1–242 and 1**–**275 proteins could be also detected in the cell membrane, we performed WB analyses using specific membrane-enrichment methods (Fig. 4D).

### GZMA, but not caspases, releases the GSDMB NT pyroptotic activity

Our data indicate that only exon 6-containing GSDMB isoforms (3 and 4) have activatable pyroptosis capability, but it is still unclear whether any protease can differentially cleave GSDMB variants and thus regulate their cytotoxic activity.

Since previous studies yielded conflicting results on the function of caspases on GSDMB pyroptotic ability [10, 29, 32], we first analyzed the effect of activating endogenous caspases in THP1 monocytes that stably over-express either GSDMB isoform 2 (1–416Δ6,7) or 3 (1–416). In these models, we induced caspase-1-triggered canonical pyroptosis (via LPS plus nigericin protocol [62]) or caspase-3-mediated apoptosis (1 μM etoposide treatment for 24 h). As shown in Supplementary Fig. 7A–C, both GSDMB isoforms are cleaved equally after caspase-1 and caspase-3 activation. The cleavage occurs within the NT region after D91 residue (which is common to all isoforms) producing a detectable CT fragment (92–416; 37 KDa). Interestingly, this caspase processing of GSDMB has no effect on cell lysis (LDH release) during LPS + nigericin induced pyroptosis (mediated by endogenous GSDMD; Supplementary Fig. 7D). Indeed, this cleavage is analogous to the caspase-3 processing on GSDMD D91 that inhibits GSDMD pyroptosis [27]. Thus, in agreement with Chen et al. [29] and Chao et al. [10], and contrary to Panganiban et al. [32], our data imply that caspases do not activate GSDMB cell death.

**Fig. 5:**
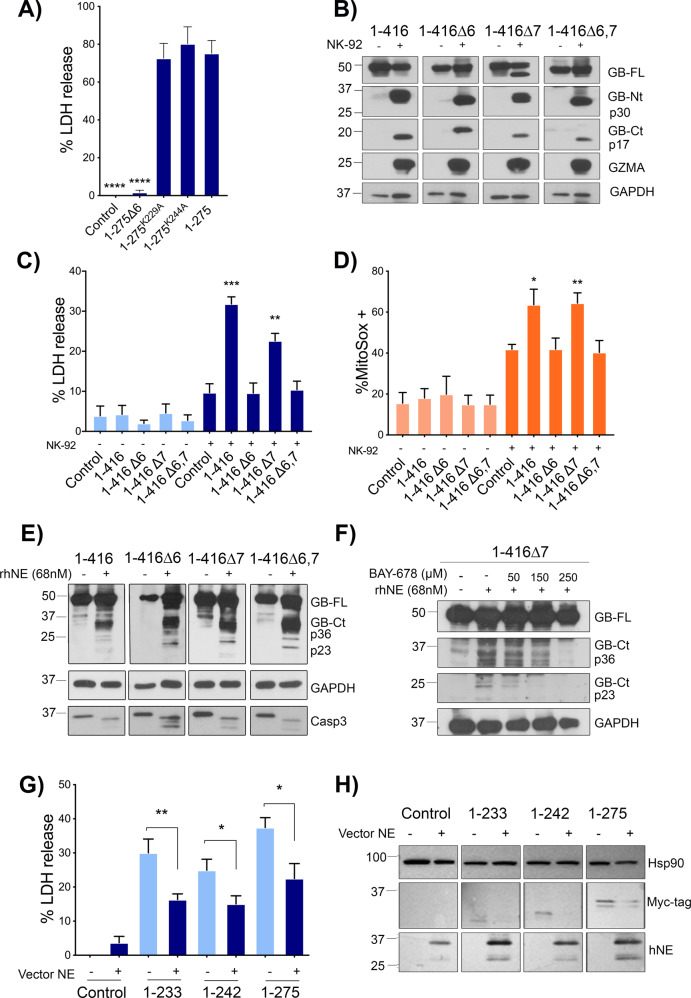
Effect of GZMA or NE cleavage on the pyroptotic activity of GSDMB isoforms. **A** Lactate dehydrogenase (LDH) assay in transiently transfected (48 h) HEK293T cells expressing 1–275 cytotoxic fragment and the corresponding individual mutants of GZMA-cleavage site 1–275^K229A^ and 1-275^K244A^. Values represent means ± SEMs of 6 independent experiments. Differences between cytotoxic 1–275 and other constructs were tested by two-tailed unpaired t-test: ****p < 0,0001; ns, not significant. **B** HEK293T cells expressing GSDMB isoforms [1–4] were co-cultured with NK-92 cells. Immunoblotting shows the cleavage of GSDMB by GZMA using anti-GSDMB-NT (Sigma, HPA023925) and anti-GSDMB-CT antibody Ab-GB [39]. **C** Lactate dehydrogenase (LDH) assay after 16 h of NK-92 co-culture and (**D**) Mitochondrial damage of HEK293T analyzed by mitoSOX using flow cytometry. NK-92 cells were labelled with CD56 antibody. LDH and MitoSOX values represent means ± SEMs of four independent experiments. Differences between control condition (HEK293T empty vector co-culture with NK-92) and each condition was tested by two-tailed unpaired t-test: *p < 0,05, **p < 0,01 and ***p < 0,001. **E** Immunoblotting analysis of GSDMB cleavage by human neutrophil elastase (rhNE). HEK-293T cell lysates containing GSDMB isoforms [1–4] were incubated with rhNE (68 nM) at 37 °C for 30 min (**F**) and in presence of indicated amount of BAY-678 inhibitor. Immunoblot probes for GSDMB cleavage detection: anti-GSDMB-NT (Sigma, HPA023925) and anti-GSDMB-CT antibody Ab-GB [39]. GAPDH was used as a loading control. **G** Lactate dehydrogenase (LDH) assay after 48 h of co-transfection of hNE vector and GSDMB constructions. Values represent means ± SEMs of six independent experiments. Differences between hNE treated and untreated conditions were tested by two-tailed unpaired t-test: *p < 0,05 and **p < 0,01. **H** HEK293T were transfected with hNE vector and subsequently with GSDMB constructs. GSDMB-NT constructs were cleaved by hNE, and this produce a decrease in the levels of GDSMB-NT fragments. Fragments were detected by anti-Myc flag located at the C-terminal region of GSDMB. HSP90 was used as a loading control.

Moreover, it has been shown that GZMA can cleave GSDMB in two Lysines, K229 (within exon 6, producing a 28 KDa NT fragment) and K244 (in exon 8, common to all GSDMB isoforms, and producing a 30KDa NT fragment), and this processing triggers GSDMB pyroptosis [17]. The K244 is the key residue, since its mutation to alanine, but not K229A, strongly abrogates GZMA-mediated GSDMB pyroptosis [17]. Here we first tested if, in addition to GZMA-cleavage, K229, and K244 were required for cell death of the 1–275 GSDMB NT cytotoxic fragment. We observed that mutating these residues to alanine did not reduce 1–275 pyroptosis, implying that these lysines are not crucial for the NT pyroptotic activity (Fig. 5A). Importantly, Zhou et al. [17] did not evaluate the differential functional effect of GZMA on the pyroptotic activation of the GSDMB transcript variants. To address this question, HEK293T cells expressing each GSDMB isoform were co-cultured for 16 h with NK-92 cells, as previously described [17]. The results confirmed the GZMA preference for the K244 cleavage site [17], since immunoblotting revealed the ∼30 kDa NT fragment in all GSDMB isoforms (Fig. 5B). Interestingly, we demonstrated that only those exon 6-containing isoforms (GSDMB3 and 4) produced substantial increase of LDH release and mitoSOX levels in HEK293 co-cultured with NK cells (Fig. 5C, D). Therefore, GZMA can cleave all GSDMB isoforms but the presence of exon 6 in the released NT fragment is required for GSDMB pyroptosis.

### GSDMB cleavage by Neutrophil elastase blocks the pyroptotic activity

Then, we studied the Neutrophil Elastase (NE), a serin protease that cleaves and activates GSDMD NT pyroptosis in specific situations [24], since our preliminary *in-silico* analysis indicated that this protease could potentially cleave GSDMB.

Indeed, the in vitro digestion of protein lysates from GSDMB-expressing HEK239T cells with recombinant human Neutrophile Elastase (rhNE) resulted in the appearance of two major CT fragments (∼36 KDa and ∼23 KDa) in all GSDMB isoforms (Fig. 5E). Further information by mass spectrometry analyses revealed that the p23 fragment resulted from the cleavage at the residue M220, while the larger p36 corresponded to the CT fragment generated by caspases [10]. Importantly, the addition of the NE specific inhibitor (BAY-678) in the in vitro reaction inhibited the generation of the p23 and not p36 in a dose-dependent manner (Fig. 5F). This demonstrates that p23 cleavage was produced by rhNE, while p36 occurred from the spontaneous processing during in vitro incubation. Remarkably, cleavage by NE at M220 would produce the 1–220 NT fragment, which we have proved before to be non-cytotoxic (Fig. 1B). Notably, NE processing can inhibit GSDMB pyroptotic activity, since transient expression of NE (before transfection with GSDMB-NT fragments) reduced the cytotoxic effects of 1–233, 1–242, and 1–275 fragments in HEK293T cells (Fig. 5G). Immunoblotting assay confirmed the reduction in the levels of NT constructs, but unfortunately the cleavage fragments could not be detected (Fig. 5H).

### GSDMB can kill cancer cells in an isoform-dependent way

**Fig. 6:**
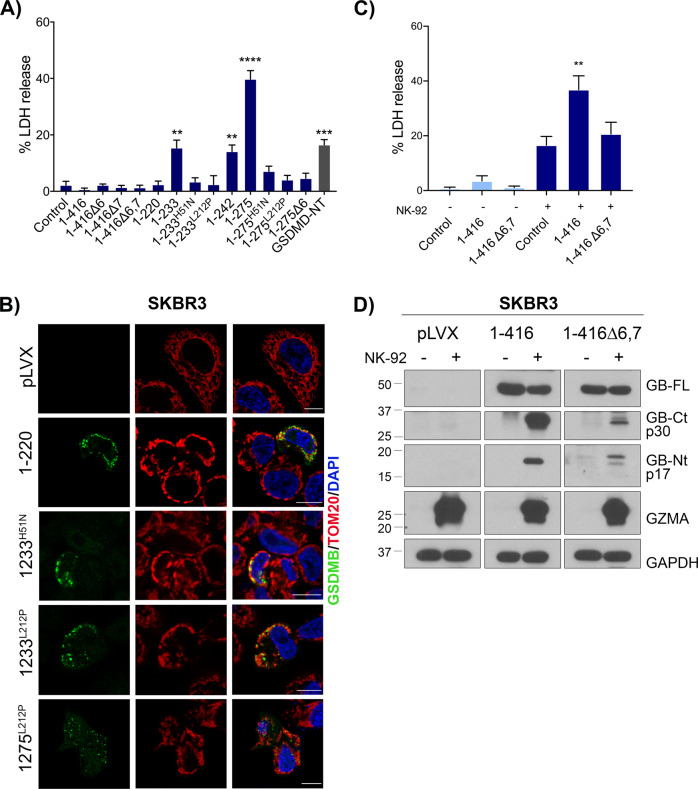
Differential cell death activity of GSDMB isoforms in breast cancer cells. SKBR3 cell line was transiently transfected with GSDMB constructs during 48 h. **A** Cytotoxicity was measured by lactate dehydrogenase (LDH) assay. Values represent means ± SEMs of 4 independent experiments. Differences between control condition (empty vector) and each condition were tested by two-tailed unpaired t-test: *p < 0,05 and **>*p* < 0,01. GSDMD-NT was used as a positive control. **B** Immunofluorescence and confocal microscopy analysis in SKBR3 cells transiently transfected with the indicated GSDMB-NT constructs. GSDMB-NT (green), colocalizes with mitochondrial marker TOM20 (red). pLVX (empty vector) was used as negative control. **C** Lactate dehydrogenase (LDH) assay after 16 h of co-culture with NK-92. SKBR3 cells expressing GSDMB isoforms 2 and 3 (1-416Δ6,7, 1-416, respectively) were co-cultured with NK-92 cells. Values represent means ± SEMs of seven independent experiments. Differences between control condition (empty vector co-culture with NK-92) and each condition were tested by two-tailed unpaired *t*-test: **p < 0,01. **D** Immunoblotting of GSDMB cleavage by GZMA in SKBR3 cells expressing GSDMB isoforms (2 and 3) cocultured with NK-92 cells. Immunoblot antibodies for GSDMB cleavage detection: anti-GSDMB-NT (Sigma, HPA023925) and anti-GSDMB-CT antibody Ab-GB [39]. GAPDH was used as a loading control.

Releasing GSDMB pyroptosis specifically in cancer cells can be a promising antitumor therapeutic approach. To assess this possibility, we first validated our results using two cancer cell models, 23132/87 (gastric cancer cell line) and SKBR3 (HER2 breast cancer) with very low or undetectable expression of GSDMB (Supplementary Fig. 1B). Replicating our results in HEK293T cells, 1–233, 1–242 and 1–275 constructs provoke pyroptotic cell death in SKBR3 (Fig. 6A) and in 23132/87 cell lines (Supplementary Fig. 8A), while the constructs lacking exon 6 or with those with point mutations (H51 or L212) again accumulated in mitochondria and produced no cell death (Fig. 6B, Supplementary Fig. 8B).

Moreover, we also confirmed that NK-released GZMA can significantly increase cell death in SKBR3 in an isoform-dependent way (Fig. 6C, D). Further corroborating the effect of NE cleavage in cancer pyroptosis, NE cleaves GSDMB in SKBR3 cells and releases the pyroptotic-inactive p23 NT fragment (Supplementary Fig. 8C).

### Differential expression of GSDMB isoforms associates with clinicopathological variables in breast cancer

**Fig. 7:**
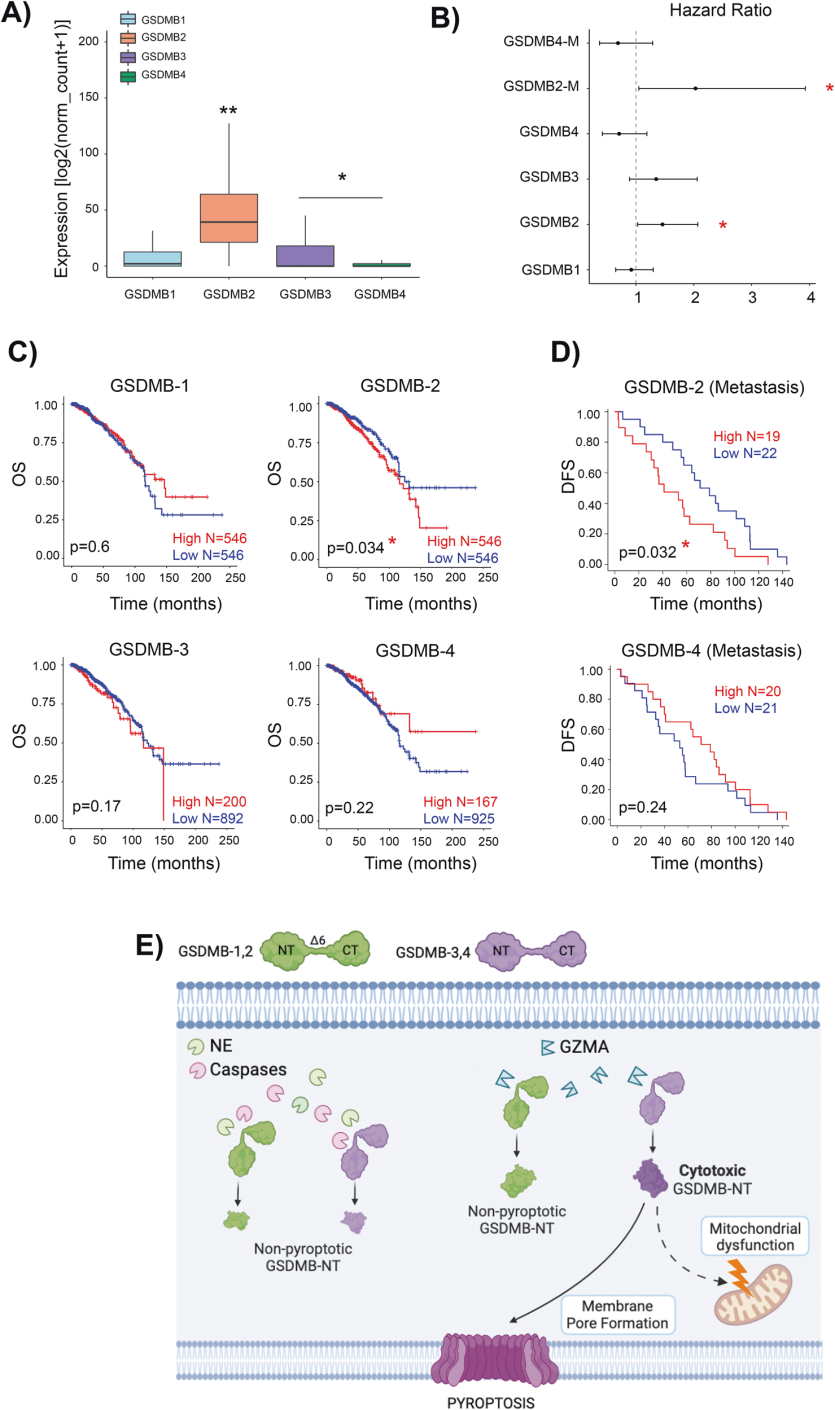
GSDMB-2 isoform expression is associated with poor prognosis in breast cancer patients. **A** Gene expression of GSDMBs isoforms from breast cancer patients (*n* = 1093) was analyzed using TCGA data. GSDMB-2 isoform was significantly overexpressed in comparison with the rest of isoforms. Statistical analyses were performed by ANOVA and Tukey comparisons: *p < 0,05 and **p < 0,01. **B** Hazard ratios were calculated by Cox proportional-hazards model in primary tumor and metastatic samples (M). **C** Overall survival (OS) analyses (log rank test) of breast cancer patients from TCGA categorized according to the level of expression of each GSDMB isoform. **D** Disease Free Survival analyses (log rank test) of metastatic breast cancer patients from the clinical trial NCT01377363. Tumors were categorized according to the expression of *GSDMB2* or *GSDMB4* (measured by real-time PCR). Survival study was performed using “survival” R package. Tumors were classified based on GSDMB isoforms expression using the mean expression value as cut-off. Results were considered significant when *p* < 0,05. **E** Schematic model representing the differential functions of GSDMB isoforms and the distinct proteases in regulating pyroptosis and mitochondrial damage. Caspases and Neutrophil Elastase (NE) generate NT fragments in all GSDMB isoforms with no pyroptotic activity. GZMA cleavage activates pyroptosis only in exon 6-containing GSDMB isoforms (GSDMB 3 and 4). Image created using Biorender ©.

Based on our results, we finally reasoned that the differential expression of *GSDMB* transcript variants could have an impact on the biological and clinical behavior of GSDMB-positive tumors, since those mostly expressing exon 6-null isoforms (*GSDMB1-2*) should not have GSDMB-mediated pyroptotic anti-tumor function. To test this hypothesis, we focus on breast tumors, since we have previously demonstrated that in mammary carcinomas, *GSDMB* over-expression has prognostic value and promotes multiple pro-tumor effects in vitro and in vivo [39, 41, 42, 48]. First, in 1093 breast cancer patients from the TCGA dataset, we observed that *GSDMB2* mean expression was generally higher than the other isoforms (Fig. 7A). Interestingly, in unselected breast carcinomas, *GSDMB2* upregulation significantly associated with reduced overall survival, while higher levels of *GSDMB1* and *GSDMB4* (alternative expression of exon 6 and 7) have the opposite effect (Fig. 7B, C). To validate these results, we assessed *GSDMB2* isoform and *GSDMB4* (with exon 6, as control) expression by qRT-PCR in 55 paired primary tumor and metastasis samples from the ConvertHER clinical trial [49] (NCT01377363). Again, we detected that *GSDMB2* expression in the metastatic lesions associated significantly with poorer overall survival. Although not significant, tumors with *GSDMB4* high expression showed better outcome (Fig. 7B, D)

These results are in line with the enhanced in vivo aggressiveness and tumorigenic potential of *GSDMB2* observed in breast cancer xenografts [48] and *GSDMB2/HER2* knock-in mouse models [41].

## Discussion

The primigenial function of GSDMs is to induce pyroptosis as protective mechanism against infection, and thus the host vs pathogen arms race could have facilitated the evolution of *GSDM* family genes in a species-specific way [63]. In this sense, human GSDMB is activated to attack intracellular *Shigella* in infected enterocytes, but mice are naturally resistant to Shigellosis and thus, lack *GSDMB* gene [64]. In addition to this antibacterial activity, *GSDMB* has evolved other cell death-dependent and independent effects, and the dysregulation of these functions can cause multiple pathologies [29, 31, 32, 35]. *GSDMB* multifunctionality may be controlled by various mechanisms, including the expression of transcriptional variants with distinct biological activities. Indeed, diverse SNPs that control the differential transcription (including exon 6 skipping) of GSDMB variants (*GSDMB1-4*) are strongly associated with inflammatory diseases [15, 37]. In cancer, previous evidences suggested that GSDMB isoforms also exhibit different effects in vivo [4]. Thus, over-expression of *GSDMB2*, but not *GSDMB1*, increases breast cancer tumor growth and metastasis in xenografted mice [48] and GEMMs co-expressing *GSDMB2* and *HER2* oncogene show enhanced breast cancer incidence [41]. By contrast, immunocyte-triggered mouse GZMA can cleave GSDMB3 in tumor xenografts leading to cancer pyroptosis [17]. However, the biological determinants underlying these functional differences among GSDMB variants remained obscure until now. Here, we prove that the translation of alternative exon 6, but not exon 7, is essential for pyroptosis, and therefore GSDMB variants lacking exon 6 (*GSDMB1-2*) are pyroptotic-deficient (Fig. 7E). Consistent with this, *GSDMB2* upregulation associates with unfavorable clinical-pathological and prognostic features in two breast cancer cohorts. These results, together with the enhanced in vivo pro-tumor effects of GSDMB2 described before [41, 48], indicate that *GSDMB2* in breast cancers not only prevents pyroptosis but mostly promotes tumor progression.

Mechanistically, the relevance of exon 6 in GSDMB pyroptosis is proved by our molecular dynamic simulation and functional studies, showing that R225, K227, K229 and E233 residues could be involved in the polymeric stability of the pore and subsequent pyroptotic induction. Moreover, our data indicate that exon 6 is not necessary for cell membrane of mitochondria localization but is required for GSDMB NT cell lysis and the concomitant mitochondrial damage.

In this sense, GSDMB NT dual impact on membrane and mitochondria mimics the effect of other GSDMs NTs (GSDMA/A3/D/E) [14, 43, 60, 65]. Recent data indicate that GSDM mitochondrial impairment generally precedes plasma membrane targeting [14, 65], but then cell membrane pores are sufficient to cause cellular lysis [66]. Depending on the GSDM member and biological context, early mitochondrial damage can activate other cooperative cell death mechanisms, like apoptosis (GSDMA and GSDME; [12, 66]), autophagic cell death (GSDMA3; [43]), or necroptosis (GSDMD; [67]). We showed that, like GSDMA and GSMDA3 NTs [43, 66], GSDMB NT mostly accumulated in mitochondria, but this did not lead to secondary apoptosis (caspase3/7 activation) or increased mitophagy (data not shown). Besides, HEK293 cells are necroptosis-deficient [68] and lack other endogenous GSDMs [17], thus GSDMB NT pyroptosis might to be self-sufficient (does not require other secondary cell death mechanisms) and involves the concomitant effect of mitochondrial damage and cell lysis. Nonetheless, since GSDM-mediated mitochondrial impairment and cell rupture proceed very quick [66] to uncover the precise intracellular kinetics and functional consequences of GSDMB activation further studies are required using additional approaches in which GSDMB NT release could be controlled in a more accurate way.

Proteases can control GSDM-mediated cell death in a complex manner, and this has been mostly studied for GSDMD in immune cells, where its cleavage by apoptotic caspases (D92 residue) inactivates pyroptosis [27], whereas NE cleavage can lead to pyroptosis, NETosis or no cell death [24, 25, 69] depending on the biological context. Here we demonstrated that GSDMB cleavage (cleavage sites are shared by all isoforms) by different peptidases have distinctive effects on pyroptosis. Hence, inflammasome-activated caspase-1 and apoptotic caspases cut GSDMB within NT (D91 site, analogous to GSDMD D92 [27]), producing GSDMB fragments with no pyroptotic activity, while NE processing at M220 significantly reduces the cytotoxic effect of pyroptotic NT fragments (Supplementary Fig. 9). This data suggest that caspases and NE might be serve as regulators for reducing GSDMB pyroptosis or for switching this lytic cell death to apoptosis in particular scenarios. In this sense, we can speculate that during immunocyte attack, released NE might preferentially trigger CD95-dependent apoptosis in cancer cells [70] and counteract GZMA-mediated pyroptosis. Importantly, we establish that GZMA preferentially cleaves all GSDMB isoforms at K244, but only exon 6-containing variants have pyroptotic activity (Supplementary Fig. 9).

It has been proposed that triggering GSDM-mediated pyroptosis in cancers could be a promising therapeutic strategy. However, GSDMB pyroptosis may depend not only on the presence/absence of specific endogenous variants and potentially inhibitory proteases, but also on the activation of specific structural mechanisms. Indeed, compared to other GSDMs, GSDMB shows distinctive features regarding protein structure and lipid binding affinity, suggesting that this protein can have exclusive mechanisms of pyroptotic regulation [10, 64]. Indeed, we observed that, contrary to other family members [8], mutating a conserved CT Alanine to Aspartic (A340 in GSDMB) does not release auto-inhibition and activate cell death. Also, GSDMB NT pyroptotic activity depends on residues that are conserved among GSDMs (like the L212) but others are unique, such as H51. Indeed, a recent structural study shows that H51 and R26 forms a basic patch that is exclusive of GSDMB (other GSDMs have acidic or polar residues in these sites) being this patch involved in lipid recognition and/or regulating pore formation [64].

Summarizing, our data contributes to clarify the mechanisms of GSDMB cell death and highlight the differential role of GSDMB isoforms in cancer biology. Hence, pyroptosis-mediated antitumor role is specific of exon6-expressing isoforms, but further studies are required to confirm if GSDMB pro-tumor functions, like cell motility [35, 42, 48] and resistance to anti-HER2 therapies [40], are common to all four isoforms.

## Supplementary information

Supplementary information

Supplementary videos 1-12

Check list

## Data Availability

The data analyzed during this study are included in this published article and the supplemental data files. Additional supporting data are available from the corresponding authors upon reasonable request.
