# Supplementary material for: Distinct GSDMB protein isoforms and protease cleavage processes differentially control pyroptotic cell death and mitochondrial damage in cancer cells
Source: Cell Death Differ. 2023 Mar 11;30(5):1366–81. doi: 10.1038/s41418-023-01143-y (PMC10154425; doi:10.1038/s41418-023-01143-y)
Supplement: Supplementary file 1 — Supplementary information [file 41418_2023_1143_MOESM1_ESM.pdf]

Supplementary Information

Distinct GSDMB protein isoforms and protease cleavage processes differentially control pyroptotic cell death and mitochondrial damage in cancer cells *by Oltra SS et al.*

Running title: Different role of Gasdermin B isoforms in pyroptosis.

Sara S Oltra, Sara Colomo, Laura Sin, María Pérez-López, Sara Lázaro, Angela Molina-Crespo, Kyoung-Han Choi, David Ros-Pardo, Lidia Martinez, Saleta Morales, Cristina González-Paramos, Alba Orantes, Mario Soriano, Alberto Hernandez, Ana Lluch, Federico Rojo, Joan Albanell, Paulino Gómez-Puertas, Jae-Kyun Ko, David Sarrió*, Gema Moreno- Bueno*

*Corresponding authors

INDEX

- Supplementary Materials/Subjects and Methods 3
- Supplementary Figures 13
- Supplementary Tables 24
- Supplementary Videos legends 27
- Uncropped Western-Blots 30
- References 39

Supplementary Materials/Subjects and Methods:

Plasmids

The pEZ-M61 plasmid with a HA flag at the C-terminal region was used to generate 1-220 and 1-275 constructs. Unfortunately, as previously described (1), 1-275 construct was highly toxic in *Escherichia coli* and could not be cloned in the pEZ-M61 plasmid that presented a bacterial T7 promoter. Furthermore, during the cloning process spontaneous mutations of the 1-275 construct arose at c.153 C>A (p.H51N) located in exon 2, and at c.637 G>A (p.L212P), located in exon 5, named as H51N and L212P, respectively. The rest of cDNA constructs were generated using the lentiviral vector pLVX with a C-terminal *myc* flag, a cytomegalovirus promotor and puromycin resistance. The cDNA was cloned in the lentiviral vector pLVX following the Ligase T4 DNA protocol (Invitogen). Mutations were generated using High-Fidelity PCR Master Mix (Thermo Fisher Scientific™, Carlsbad, CA, USA). The pEZ-M61 vector was obtained from Genecopoeia (Rockville, MD, US), construct pLVX from Takara (Takara Bio, USA) and Neutrophile Elastase cloned in the pCMV6-XL4 from Origene (Rockville, MD, US). Inducible Lentiviral Vector tagged with GFP at the C-terminal was used for the inducible GSDMB fragments (Inducible Lenti-TRE3G-ORF-C-TagGFP2-PGK-Tet3G-puro; from Transomic, Supplementary **Table 2**).

Transformation was carried out in DH5α bacteria following the conventional thermal shock transformation protocol. cDNA was isolated by High Pure Plasmid Isolation Kit (Roche) and all plasmids were verified by DNA sequencing. As additional controls, GSDMD full-length cDNA (pCS2-3XFlag-hGSDMD) and GSDMD-NT (pCS2-3XFlag-hGSDMD 1-275) were kindly provided by Dr. Pablo Pelegrin (Instituto Murciano de Investigación Biosanitaria Virgen de la Arrixaca, IMIB, Murcia, Spain).

Cell culture and transient transfection

HEK293T cells were grown in Dulbecco's modified Eagle's medium (DMEM) supplemented with 10% fetal bovine serum (FBS) and 2 mM L- glutamine. SKBR3 and 23132/87 cell lines

were grown in RPMI medium supplemented with 10% FBS and 2 mM L- glutamine. NK-92 cells were growth in 75% alpha-MEM supplemented with 12.5% FBS, 12.5% of horse serum, 2 mM of L-glutamine and 5ng/ml of IL-2. THP1 cells were cultured in RPMI medium supplemented with 10% FBS, 1% streptomycin/penicillin, 1% glutamine and 0.5% fungizone. All cells were grown at 37°C and 5% of CO_2. For co-culture assays, target HEK293T or SKBR3 cells were seeded in 12-well plates (3 x 10^5 cell/well) and transfected for 48h. Then, NK-92 cells were added at 8:1 ratio (Effector cell: Target cell) and co-cultured for 16h. Detailed information about culture conditions has been included in **Supplementary Table 1**. Cell lines were purchased from commercial repositories (**Supplementary Table 2**) and their identity was confirmed by short tandem repeat profiling. Cells were routinely tested for Mycoplasma infection.

Transient transfection of all constructs and corresponding empty vectors were performed using lipofectamine 2000 (Invitrogen) according to the manufacturers' protocol. For those cells transfected with the constructs cloned into Doxycycline-inducible lentiviral vectors, we added Doxycycline at 200ng/ml to induce gene expression at intended time frames. Regarding the neutrophile elastase assay, cells were firstly transfected with NE and 24h later with GSDMB constructs.

Lactate dehydrogenase (LDH) cytotoxicity assay

LDH release was assayed using the Cytotoxicity Detection KitPLUS LDH (Roche) following the manufacturer's protocol. Culture media from transfected cells, as indicated previously, was collected and centrifugated at 1 200 rpm, 4°C for 5 min to remove cell debris. LDH release was measured at OD 490. The percentage of LDH release, indicative of cytotoxicity, was calculated as follows: (experimental value – lower value)/ (positive control – lower value) x 100. As positive control (maximum release) we lyse cells with Triton X-100.

MitoSOX and TMRE assays

MitoSOX Deep red fluorescent probe (Thermo Fisher Scientific™) was used to determine the superoxide production by the GSDMB constructs in cell lines. Briefly, superoxide levels were

measured by mitoSOX in transiently transfected cells (48 h). After removal of transfection medium cells were incubated in 5µM mitoSOX for 30 min at 37°C. The mitoSOX levels were assayed by flow cytometry using a FACSCanto II flow cytometer (Becton Dickinson). Mitochondrial transmembrane potential was measured using tetramethylrhodamine ethyl ester (TMRE, abcam) in transient transfected HEK293T cells. Cells were incubated with 500nM of TMRE for 15min at 37°C. As a positive control for depolarizing mitochondrial membrane potential, cells were previously incubated with 20µM of FCCP (carbonilcyanide p-triflouromethoxyphenylhydrazone) for 10min at 37°C. After incubation, red fluorescence was detected in PBS/0,2% BSA by a fluorescence plate reader (Promega) at Ex/Em: 549/575nm.

Mitochondria purification from cell lines

Mitochondrial isolation method was done following the protocol detailed in (2). Briefly, 9 x 10^6 HEK293T cells were culture in 150 mm plates at 80-90% confluency and transfected with different GSDMB constructs during 48h. Cells were harvested by cell scrapper, pelleted at 600g for 5 min and washed twice in PBS. The cell pellets were placed on ice and broken by adding one volume of hypotonic homogenization buffer (IB 0.1x: 3.5 mM Tris–HCl, pH 7.8, 2.5 mM NaCl, 0.5 mM MgCl2), and homogenized by 10 strokes using a Thomas homogenizer with a motor-driven Teflon pestle. Immediately after, 1/10 of the packed cell volume of hypertonic buffer was added to make the medium isotonic. Homogenate was centrifuged at 1200g for 3 min at 4 °C to pellet unbroken cells, debris, and nuclei. Supernatant was collected and centrifuged again at low speed in the same conditions (1200g for 3 min at 4 °C). Mitochondria contained on the supernatant were pelleted in Eppendorf tubes (adding approximately 1 ml of supernatant per tube) by centrifugation in microfuge at 15 000 g during 2 min at 4°C. Pellets were washed using homogenization buffer A (0.32 M sucrose, 1 mM EDTA, and 10 mM Tris–HCl, pH 7.4) and again, resuspended in the appropriate buffer and kept at 4 ºC until used.

Mitochondrial DNA release assay

Human mitochondrial DNA was isolated from the cytosolic fraction of transfected HEK293T with different GSDMB constructs using the DNeasy Blood &Tissue Kit (QIAGEN). The standard of mitochondrial DNA was obtained from mitochondria isolated from HEK293T cells using the protocol previously described in (2). Mitochondrial DNA standard curve was obtained in each assay for their absolute quantification. Quantitative PCR was employed to measure mitochondrial DNA using Power SYBR® Green PCR Master (Applied Biosystems) in a StepOnePlus™ Real-Time PCR System, using the following primers: Cytochrome c oxidase I (Forward: 5'-GCCCCAGATATAGCATTCCC-3' and reverse: 5'-GTTCATCCTGTTCCTGCTCC-3') and 18S rDNA (internal control) (Forward: 5'-TAGAGGGACAAGTGGCGTTC-3' and reverse: 5'-CGCTGAGCCAGTCAGTGT-3') (3).

Pyroptosis and apoptosis assays in THP1 cells

The human non-adherent monocyte-like cell line THP1 were infected with lentiviral particles containing the following myc-tagged GSDMB plasmid constructs: 1-416, 1-416Δ6,7 or empty pLVX plasmid (as control). Stable expressing cells were maintained in the presence of the selection antibiotic, puromycin (12.5 μg/μL). For inducing canonical pyroptosis, $1x10^7$ cells were cultured in P100 plates with 6 mL RMPI medium in the absence of puromycin. To differentiate THP1 cells into macrophages, 0.08 μL/mL of PMA (forbol-12-miristate-13-acetate; SIGMA) were added and incubated for 24 h. Then, medium was replaced and 1 μL/mL of LPS (Lipopolysaccharide, SIGMA). After 24 hours, medium was replaced with RPMI without phenol red and containing 3 μL/mL of Nigericin (Cayman) to induce pyroptotic cell death. Control cells did not have LPS nor Nigericin. After 4 hours, medium containing dead cells was collected and centrifuged 5 minutes at 1200 rpm. The cell pellet was combined with the cells adhered to the plate, for subsequent protein extraction, as described in "protein extraction method". The supernatant was stored at -80ºC. To quantify pyroptosis, the enzymatic activity of the released LDH (Lactate Dehydrogenase) was measured from 1 mL of the supernatant with the Cytotoxicity

Detection KitPLUS (LDH) (Roche). For apoptosis induction, cells were treated with either 1 μL/mL of 10 μM etoposide (SIGMA) or DMSO, as control. After 24 hours, the supernatant containing dead cells was processed as described in the pyroptosis assays for Western blotting.

Neutrophil Elastase protease assay

Transient transfected cells were lysed in 0,1% Triton X-100 lysis buffer without protease inhibitors and subsequently sonicated twice during 30s (Soniprep 150). Lysates were centrifugated at 10 000 g for 10 min at 4°C and the supernatant was collected to determine the protein concentration by BCA assay (Thermo Fisher Scientific™). Cell lysates (12,5 ug for hNE) were mixed at indicated concentrations of recombinant human neutrophile elastase (Sigma), followed by incubation at 37°C for 1h. Elastase reactions were carried out in the lysis buffer containing 0,1% Triton X-100. Where indicated, BAY-678 inhibitor was incubated with the protease treatment at the same conditions. Reactions were stopped by adding 1x Leammli Buffer with DTT (25mM) and incubated for 5 min at 95°C prior to loading on SDS-PAGE.

Western Blot

Total proteins were extracted in lysis buffer (0,1M NaCl, 0,05M Tris HCl pH 7,9, 5μM MgCl2, 5μM CaCl2 and 2% SDS) containing protease and phosphatase inhibitor cocktail (2mM PMSF, 2μg/ml leupeptin, 20μg/ml aprotinin, 1mM sodium orthovanadate, 5mM NaF y 5mM b-glycerophosphate; Sigma Aldrich). After 30s sonication (Soniprep 150) protein was quantified by BCA assay (Thermo Fisher Scientific™). Membrane proteins were collected following the Plasma Membrane Protein Kit protocol (Abcam, ab65400). HE293T cells were transfected with different GSDMB constructs during 48h. Both, the cytosolic and membrane fractions were lysate in 0,5% Triton in PBS. Laemmli 5X buffer was finally added. Proteins were resolved in 10 or 12% SDS-PAGE and transferred on nitrocellulose membranes. After blocking the membranes with 5% skimmed milk in TBS 1X-Tween (0,01%) for 1h, primary antibodies (listed in **Supplementary Table 2)** were incubated overnight at 4°C. After washing, secondary antibodies

were added at 1:3 000 for 1h at RT. Immunoblots were detected with Pierce ECL Western Blotting Substrate (Thermo Fisher Scientific™). Uncropped western blots are included in **Supplementary Information**.

Apoptosis Caspase 3/7 detection by cytometry

CellEvent™ Caspase 3/7 Green Flow Cytometry Assay Kit (Thermo Fisher Scientific™) was used to detect apoptosis in HEK239T cells transfected during 48h with different GSDMB constructs. Additionally, the kit includes the SYTOX® AADvanced™ which detect death cells allowing the identification of viable cells and necrotic cells. Apoptotic, necrotic, and viable cells were measured by flow cytometry using a FACSCanto II flow cytometer (Becton Dickinson).

Cell death Annexin/ PI detected by flow cytometry

Cell death analysis was performed using FITC Annexin V Apoptosis Detection Kit (Immunostep,) following the manufacturer's protocol. HEK293T cells were transfected with GSDMB constructs during 24h. Cells were analyzed by FACSCanto II flow cytometer (Becton Dickinson). The percentage of double-positive (Annexin-V+, PI +) cells was quantified and compared with control cells (empty vector).

Immunofluorescence, confocal microscopy and Cell Observer

Cells were seeded in 24-well cell culture plate (1,5 x 10^5 cell/well) and transfected for 72 hours. Then cells were washed with PBS and incubated with red MitoTracker™ Deep Red FM (Thermo Fisher Scientific™) for 30 min at 37°C. Next, cells were fixed with 4% paraformaldehyde 20 min at room temperature. After 15 min of permeabilization with Triton X100 0,1%, cells were washed with PBS and incubated with primary antibodies (detailed in **Supplementary Table 2)** for 90 min at room temperature. Then, secondary antibodies were incubated for 45 min at 1:1 000. Immunofluorescence preparations were visualized in a confocal microscopy LSM710 (Zeiss) and images were processed by Fiji software (Image J 1.52).

To assess GSDMB intracellular localization in real time, HEK293T cells were transiently transfected with Doxycycline-inducible vectors expressing GFP-tagged GSDMB constructs. Cells were pre-induced with Doxycycline at 200ng for 2h. Additionally, to assess the dynamics of cell death induction, cells were cultured in the presence of 0.2µg/ml Propidium Iodide. Live videos (1 frame every 10 minutes for at least 20h) were recorded with either Cell Observer Microscopy or LSM710 confocal microscopy (Zeiss). Videos were processed by Microscope Software ZEN lite (Zeiss) and Fiji software (Image J 1.52), respectively.

Correlative light and electron microscopy (CLEM)

For correlative light and electron microscopy (CLEM) studies in 23132/87 cells were seeded in a permanox Lab-Tek chamber slide of 4 wells (Nalge Nunc International) at a density of 1,5 x 10^5 cell/well and transfected with doxycycline inducible vectors 1-220-GFP and 1-242-GFP as previously mentioned. After 6h of transfection, cells were induced with Doxycycline at 200ng/ml. Cells were incubated with red MitoTracker™ Deep Red FM (Thermo Fisher Scientific™) for 30 min at 37°C. Finally, the cells were fixed in 3 % glutaraldehyde in 0.1 M phosphate buffer (PB) for 1 hour at 37ºC. Last, were washed in 0.1 M phosphate buffer (PB) for 5 times and stored at 4 ºC.

The slides were assembled with Dako Mounting Medium containing DAPI. The confocal images were acquired with a Leica TCS SP8 HyVolution II (Leica Microsystems) inverted laser scanning confocal microscope using oil objective 63X Plan-Apochromat-Lambda Blue 1.4 N.A. The excitation wavelengths for fluorochromes were 488 nm for GFP, 638 nm for Mitotracker Deep Red and 405 nm for Dapi. Optical sections were acquired every 0.346 µm. Two-dimensional pseudo color images (255 color levels) were gathered with a size of 1024x1024 pixels, 2X optical zoom and Airy 1 pinhole diameter. After fluorescence capture, slides were processed for transmission electron microscopy analysis. The samples were postfixed in 2% OsO_4 for 1h at room temperature and stained in 2% uranyl acetate in the dark for 2h at 4 °C. Then, were rinsed in distilled water, dehydrated in ethanol, and infiltrated overnight in Durcupan resin (Sigma-Aldrich). Following polymerization, embedded cultures were detached from the wells and glued

to Durcupan blocks. Finally, ultrathin sections (0.08 µm) were cut with an Ultracut UC-6 (Leica microsystems), stained with lead citrate (Reynolds solution) and examined under a transmission electron microscope FEI Tecnai Spirit BioTwin (Thermo Fisher Scientific™). Pictures were taken using Radius software (Version 2.1) with a Xarosa digital camera (EMSIS GmbH).

Structural modeling

Structural model of the monomeric form of N-terminal of both 1-275 and 1-275Δ6 human GSDMB protein after proteolytic cleavage were obtained using procedures of homology modeling (4), using as templates the cryo-EM structures of the membrane pore formed by the N-terminal domains of murine Gasdermin A3, Protein Data Bank (PDB) accession number 6CB8 (5) and human Gasdermin D, PDB accession number 6VFE (6). The model of the dimer formed by two N-terminal domains was constructed using consecutive monomers belonging to these same membrane pore structures as a template.

Models were subjected to 100ns of unrestrained molecular dynamics (MD) simulations using the AMBER18 molecular dynamics package (http://ambermd.org/; University of California), essentially as previously described (7). 3D structures were solvated with a periodic octahedral pre-equilibrated solvent box using the LeaP module of AMBER, with 12 Å as the shortest distance between any atom in the protein subdomain and the periodic box boundaries. MD simulation was performed using the PMEMD program of AMBER18 and the ff14SB force field (http://ambermd.org/), applying the SHAKE algorithm, a time step of 2 femtoseconds (fs) and a non-bonded cut-off of 12Å. Systems were initially relaxed over 10,000 steps of energy minimization, using 1,000 steps of steepest descent minimization followed by 9,000 steps of conjugate-gradient minimization. Simulations were then started with 20 picoseconds (ps) heating phase, raising the temperature from 0 to 300 K in 10 temperature change steps, after each of which velocities were reassigned. During minimization and heating, the Cα trace dihedrals were restrained with a force constant of 500 kcal mol^{-1} rad^{-2} and gradually released in an equilibration phase in which the force constant was progressively reduced to 0 over 200 ps. After the equilibration phase, 100 ns of unrestricted MD simulation were obtained, continuously

monitoring the changes in the structure during the procedure. Figures were generated using the Pymol Molecular Graphics System.

qRT-PCR in human samples

Gene expression was analyzed by real-time PCR, using StepOnePlus Real-time PCR System, with TaqMan® Fast Advanced Master Mix (Applied Biosystems™ by Thermo Fisher Scientific™). Normalization was done with *GAPDH*. Relative expression was calculated using the comparative Ct method and obtaining the fold-change value (ΔΔCt). TaqMan probes used for qPCR assessment of *GSDMB* expression and *GSDMB* isoforms are listed in **Supplementary Table 2**.

In-Gel Digestion and Reverse phase-liquid chromatography RP-LC-MS/MS analysis

After drying, gel bands or spots were distained in acetonitrile:water (ACN:H2O, 1:1), were reduced and alkylated (disulphide bonds from cysteinyl residues were reduced with 10 mM DTT for 30 min at 56 ºC, and then thiol groups were alkylated with 10 mM iodoacetamide for 30 min at room temperature in darkness) and digested in situ with sequencing grade trypsin (Promega) as described by (8) with minor modifications (9). The gel pieces were shrunk by removing all liquid using sufficient ACN. Acetonitrile was pipetted out and the gel pieces were dried in a speedvac. The dried gel pieces were re-swollen in 100 mM Tris-HCl pH 8, 10mM $CaCl_2$ with 12.5 ng/µl trypsin for 1h in an ice-bath. The digestion buffer was removed, and gels were covered again with 100 mM Tris-HCl pH 8, 10mM $CaCl_2$ and incubated for 12 h at 37°C. Digestion was stopped by the addition of 1% TFA. Whole supernatants were dried down and then desalted onto ZipTip C18 Pipette tips (Millipore) until the mass spectrometric analysis. The desalted protein digest was dried, resuspended in 10 µl of 0.1% formic acid and analyzed by RP-LC-MS/MS in an Easy-nLC II system coupled to an ion trap LTQ-Orbitrap-Velos-Pro hybrid mass spectrometer (Thermo Fisher Scientific™). The peptides were concentrated (on-line) by reverse phase chromatography using a 0.1mm × 20 mm C18 RP precolumn (Thermo Fisher Scientific™), and then separated using a 0.075mm x 250 mm C18 RP column (Thermo Fisher Scientific™)

operating at 0.3 µl/min. Peptides were eluted using a 60-min dual gradient. The gradient profile was set as follows: 5−25% solvent B for 68 min, 25−40% solvent B for 22 min, 40−100% solvent B for 2min and 100% solvent B for 18 min (Solvent A: 0,1% formic acid in water, solvent B: 0,1% formic acid, 80% acetonitrile in water). ESI ionization was done using a Nano-bore emitters Stainless Steel ID 30 µm (Proxeon) interface at 2.1 kV spray voltage with S-Lens of 60%. The Orbitrap resolution was set at 30 000 (10).

SUPPLEMENTARY FIGURES

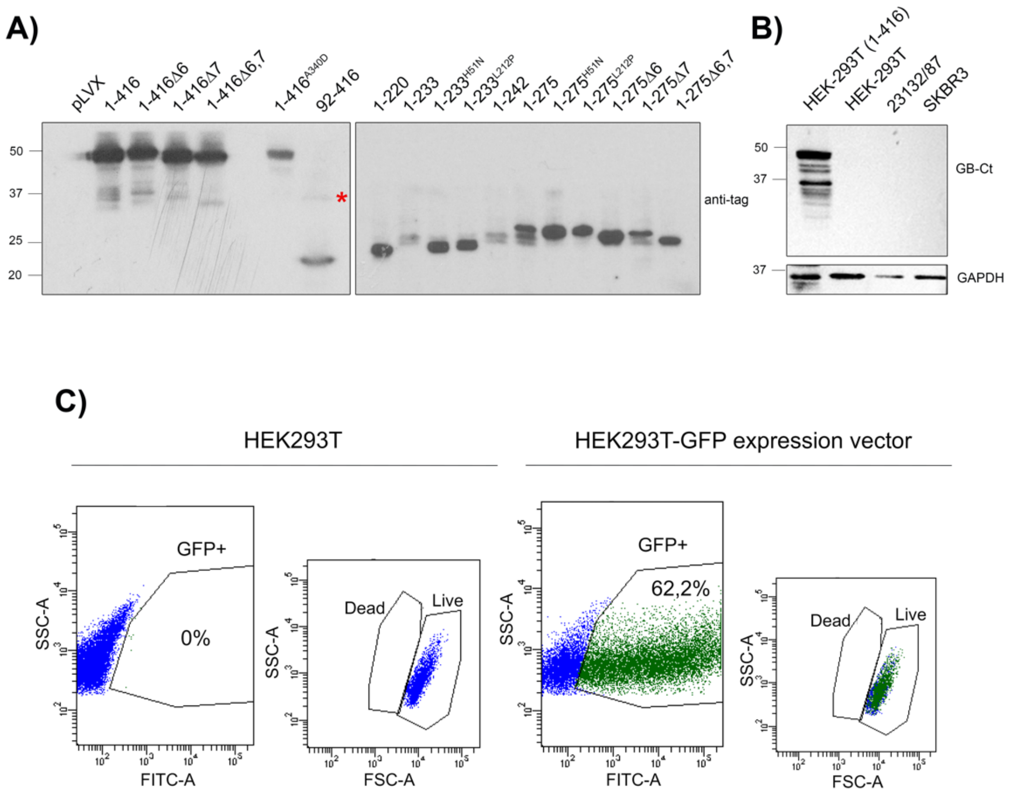

Supplementary Figure 1. A) Western blot representation of all constructs included in the study. GSDMB constructs were transiently transfected during 48 h in HEK293T cell lines. Protein expression was analyzed by Western Blot using anti-GSDMB detecting CT region antibody Ab-GB (11) or the anti-myc or anti-HA antibody detecting the tag from the CT. *Band expected for the construct 92-416. **B**) Western blot showing GSDMB endogenous protein expression in the different cell lines. HEK293T transiently transfected with GSDMB-full length (1-416) was used as a positive control. GSDMB was detected using anti-GSDMB-CT antibody Ab-GB (11). **C**) Transfection efficiency of GFP expression vector in HEK293T (right panel) compared with non-transfected cells (left panel). Percentage of GFP+/transfected cells was analyzed by flow cytometry.

A)

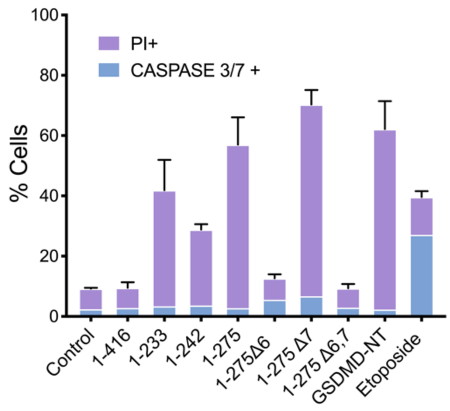

B)

```
GSDMA    -MTMFENVTRALARQLNPRGDLTPLDSLIDFKRFHPFCLVLRKR-KSTLFW-GARYVRTD    57
GSDMB    MFSVFEEITRIVVKEMDAGGDMIAVRSLVDADRFRCFHLVGEKR----TFF-GCRHYTTG    55
GSDMC    MPSMLERISKNLVKEIG-SKDLTPVKYLLSATKLRQFVILRKKKDSRSSFWEQSDYVPVE    59
GSDMD    MGSAFERVVRRVVQELDHGGEFIPVTSLQSSTGFQPYCLVVRKP-SSSWFW-KPRYKCVN    58
GSDME    ---MFAKATRNFLREVDADGDLIAVSNLNDSDKLQLLSLVTKKK--RFWCWQRPKYQFLS    55
PJVK     ---MFAAATKSFVKQVGDGGRLVPVPSLSEADKYQPLSLVVKKK--RCFLFPRYKFTSTP    55
                                                            H51N

GSDMA    EVTLERAGKAE-ACFSLPFFAPLG---LQ----GS-INHKEAVTIPKGCVLAFRVRQLMV    212
GSDMB    EETLKSDR-------QYKFWSQIS-----QGHLSYKHKGQREVTIPPNRVLSYRVKQLVF    214
GSDMC    NTVLYDSSSVN-ILGKIALWITYG---KGQGQGESLRVKKKALTLQKGMVMAYKRKQLVI    222
GSDMD    EVEVTRTHKRE-GSGRFSLPGATC----LQGEGQGHLSQKKTVTIPSGSTLAFRVAQLVI    223
GSDME    KCVISEHMQVEEKCGGIVGIQTKTVQVSATEDGNVTKDSNVVLEIPAATTIAYGVIELYV    226
PJVK     QCSLSVHAGIRGEAM----------RFHFM-DEQNPKGRDKAIVFPAHTTIAFSVFELFI    216
                                                             L212P

GSDMA    KGKDE----------------WDIPH---ICNDNMQTFPP--------------------    233
GSDMB    PNKETMN-IHFRGKTKSFPEEKDGAS---SCLGKSLGS----------------------    248
GSDMC    KEKAI-L-ISDDDEQRTFQDEYEISEMVGYCAARSEGLLPSFHTISPTLF-NASSNDMKL    279
GSDMD    DSDLDVL-LFPDKKQRTFQPPA-----TGHKRSTSEGAWPQLPSGLS----------M--    265
GSDME    KLDGLFEFCLLRGKQGGFENKKRIDSVYLDHLVFREFAFIDMPDAAHGISSQDGPLSVLK    286
PJVK     YLDGAFDLCVTSVSKGGFEREETATFALLYRL--RNILFER----------NRRVMDVIS    264
                    R225A K227A K229A E233A

GSDMA    EVTLEALPKDV-LLSKEAVGAILYFVGALTELSEAQQKLLVKSMEKKILPVQLKLVESTM    370
GSDMB    KPLLSSLFNAAGVLVEARAKAILDFLDALLELSEEQQ-FVAEALEKGTLPLLKDQVKSVM    371
GSDMC    GAILKKLQQDSNHAWFNPKDPILYLLEAIMVLSDFQHDLLACSMEKRILLQQQELVRSIL    434
GSDMD    GAVLECLVLSSGMLVPELAIPVVYLLGALTMLSETQHKLLAEALESQTLLGPLELVGSLL    409
GSDME    DAGSKQLFMT-----------AYFLVSALAEMPDSAAALLGTCCKLQIIPTLCHLLRALS    424
PJVK     ----KYVRLH-------------------------------------AVPCFDIWHKRMK    352
                                A340D
```

Supplementary Figure 2. A) Apoptosis assays by flow cytometry analyses of caspase3/7 (light blue) activation (CellEvent™ Caspase 3/7 Green Flow Cytometry Assay Kit) and Sytox Green cell dye (purple). As positive control for apoptosis, cells were treated with etoposide (25µM) for 24h. Values represents the percentage of cells positive for each condition from four independent experiments. **B**) Sequence alignment of the six GSDMs members. Point GSDMB mutation positions are indicated in different colors for all GSDMs.

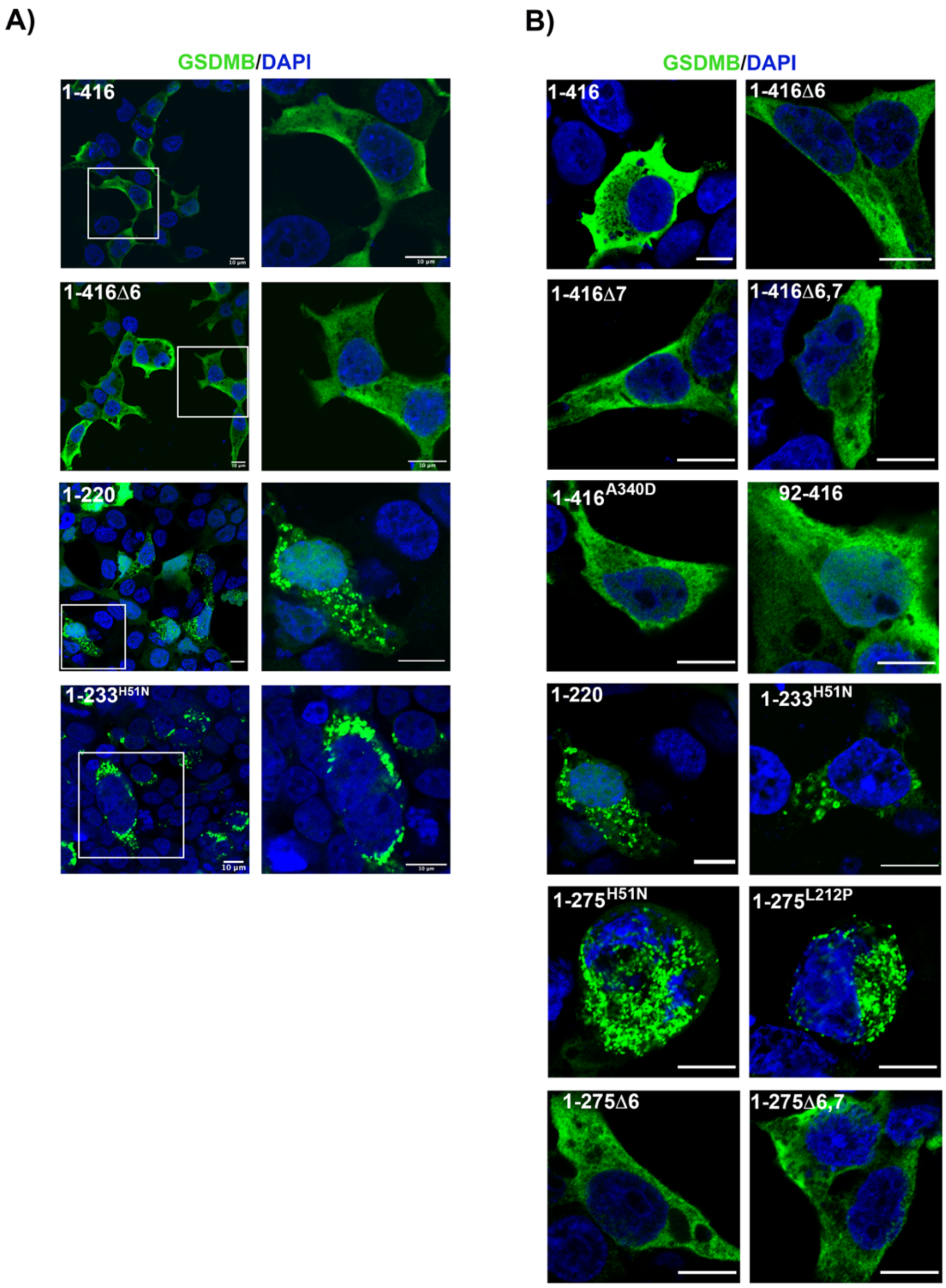

Supplementary Figure 3. A) Subcellular localization of GSDMB constructs in HEK293T cells transiently transfected during 48h. Wide field images (left) show various cells strongly positive for GSDMB-fragments, and the selected area (marked by white square) is visualized as a zoomed image on the right. **B**) Subcellular localization of GSDMB constructs in HEK293T cells transiently transfected during 48h. Localization was analyzed by immunofluorescence and confocal microscopy. GSDMB NT (green) was detected with SIGMA antibody (HPA023925). Nuclei (blue) was stained with DAPI. Scale bar represents 10µm.

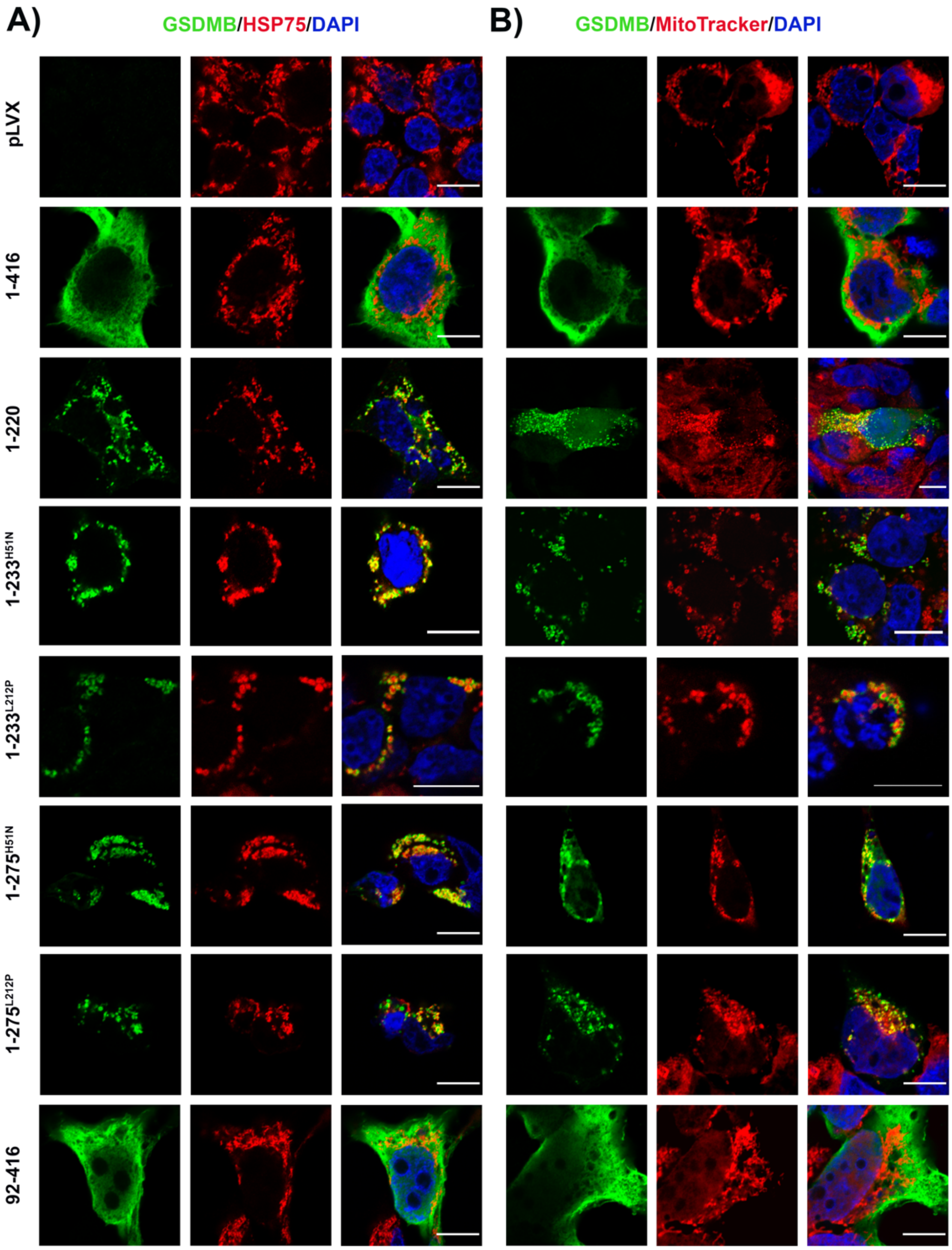

Supplementary Figure 4. GSDMB-NT aggregates co-localize with mitochondrial markers Hsp75/Trap1 and MitoTracker. HEK293T cells were transiently transfected (48h) with the indicated GSDMB constructs. Intracellular localization of GSDMB constructs (green; NT antibody SIGMA, HPA023925) and co-localization with mitochondria (red) Hsp75 (**A**) and

MitoTracker Deep Red (**B**) by immunofluorescence and confocal microscopy analysis. Cells transfected with empty vector (pLVX) were used as a negative control. GSDMB-Full length protein (1-416) and CT region (92-416) do not co-localize with mitochondrial markers. Nuclei (blue) was stained with DAPI. Scale bar represents 10μm.

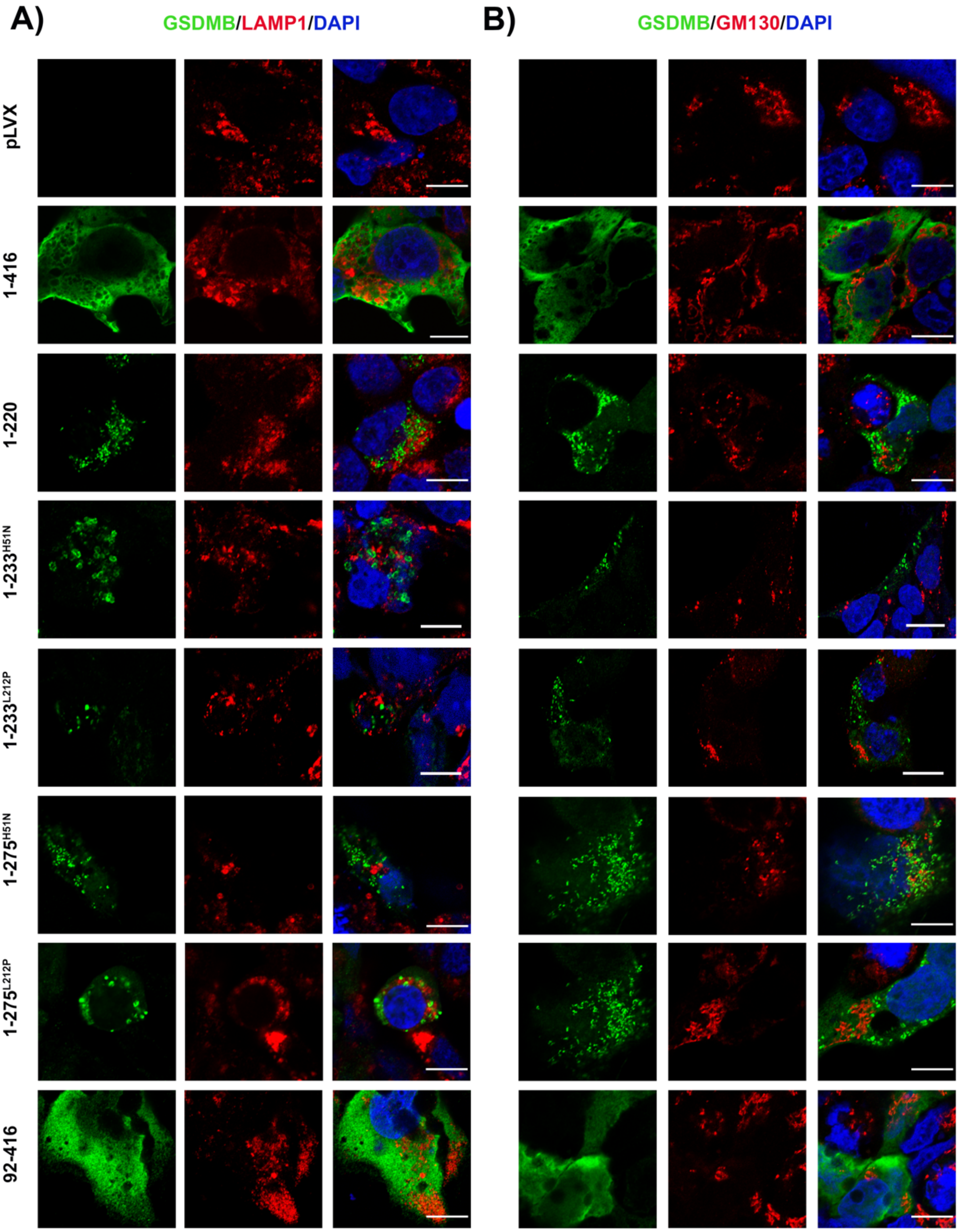

Supplementary Figure 5. GSDMB-NT aggregates do not co-localize with lysosome (LAMP1) or Golgi (GM130) markers. HEK293T cells were transiently transfected (48h) with the indicated GSDMB constructs. Intracellular localization of GSDMB constructs (green; NT antibody SIGMA, HPA023925), lysosomes (red, LAMP) (**A**) and Golgi (red, GM130) (**B**) by

immunofluorescence and confocal microscopy analysis. Nuclei (blue) was stained with DAPI. Scale bar represents 10µm.

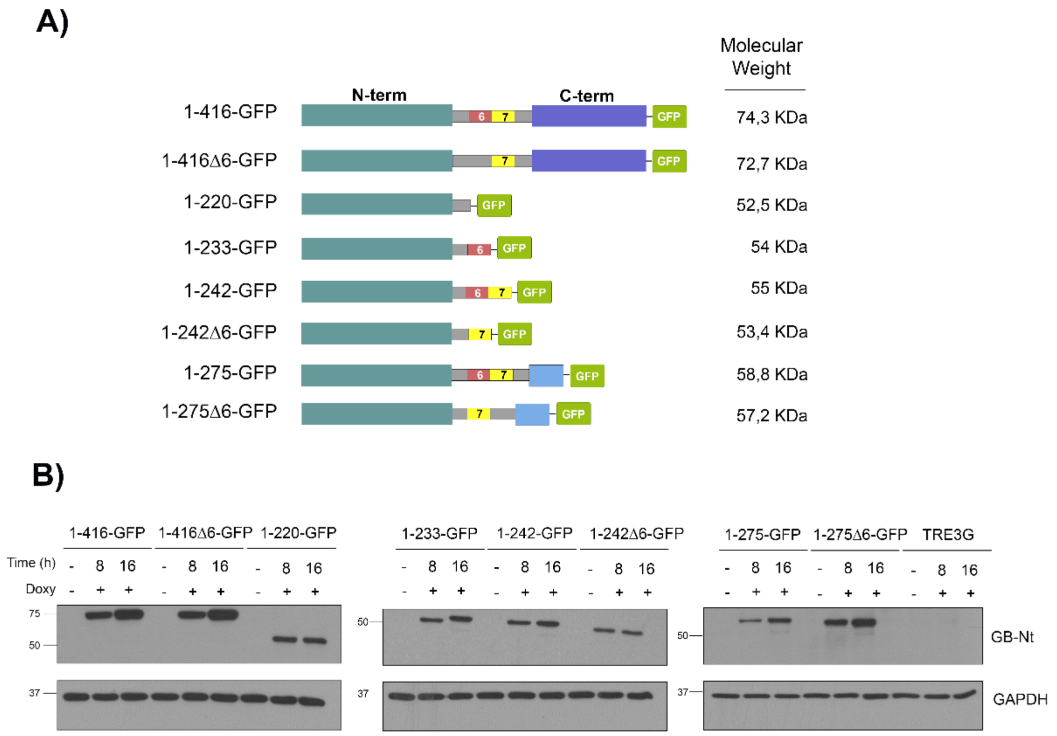

Supplementary Figure 6. Doxycycline inducible GSDMB constructs fused (in the CT) with green fluorescence protein (GFP). **A**) Schematic representation of the constructs. **B**) Western-blot confirming the inducible expression of GSDMB-GFP constructs. HEK293T were transiently transfected (24h) and GSDMB-GFP was induced by Doxycycline at 200ng/ml during 8 or 16h. GSDMB-GFP constructs were detected using anti-GSDMB detecting NT region (Santa Cruz Biotech, sc-101239). GAPDH was used as a loading control.

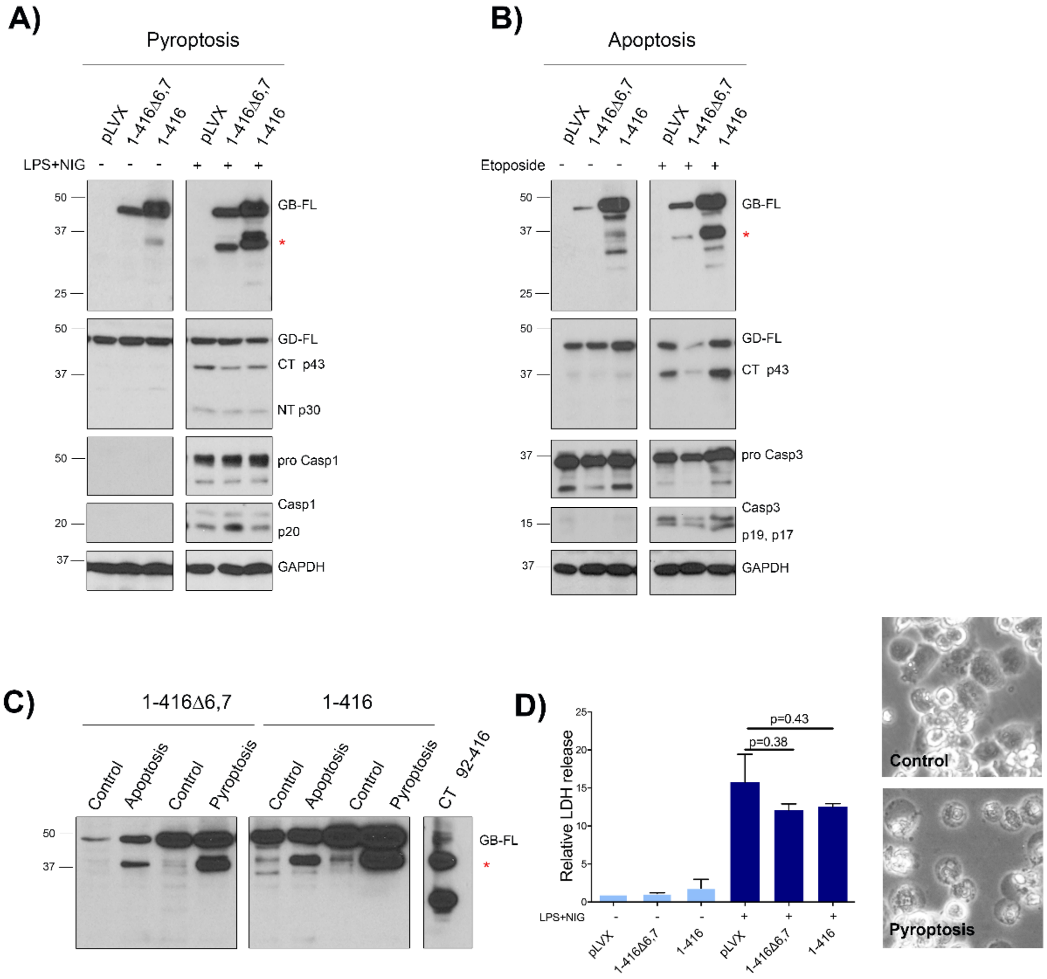

Supplementary Figure 7. GSDMB is cleaved in a similar way during pyroptosis and apoptosis and its over-expression does not affect the extent of pyroptosis in THP1 cells.

A-B) THP1 stably expressing either GSDMB isoform 2 (1-275Δ6,7), isoform 3 (1-416) or the empty vector (pLVX) were induced to undergo pyroptosis (**A:** 24h LPS and 4h Nigericin) or apoptosis (**B:** 10μM etoposide or DMSO for 24h) and cell lysates were subject to Western Blot. Using an anti-GSDMB-CT antibody Ab-GB (11) a CT fragment (asterisk) of 35 KDa (1-275D6,7) and 37 KDa (1-416) was detected in both pyroptosis and apoptosis assays. For comparison, GSDMD showed a differential cleavage pattern between apoptosis (p43 CT fragment) and pyroptosis (p30 NT fragment and secondary p43 CT fragment), as reported before (12). Pro-caspase 1 and activated caspase-1 were detected in the supernatant from pyroptotic cells (**A**). Caspase 3 activation was detected in cell lysates undergoing apoptosis (**B**). GAPDH was used as

a loading control. Molecular weights are shown on the left. **C)** Comparison in parallel of cell lysates from the pyroptosis and apoptosis experiments shown in (**A-B**) for each GSDMB isoform, confirming that the detected CT fragment (asterisk) equals to the 92-416 construct (which corresponds to the CT peptide generated by caspases 1/3/4/7/8/9 (13, 14). **D)** No significant effect of GSDMB isoform over-expression on the extent of pyroptotic cell lysis measured by LDH release. Canonical pyroptosis was induced by LPS and Nigericin treatment. Control cells were grown under standard culture conditions. Bars represents mean values ± SD from three independent experiments. Differences between control (pLVX) and GSDMB-expressing conditions were tested by two-tailed unpaired t-test. Phase contrast photographs taken of control treated cells (top) and LPS+Nigericin treated cells (bottom). Note the changes on morphology and adhesion, typical of pyroptosis.

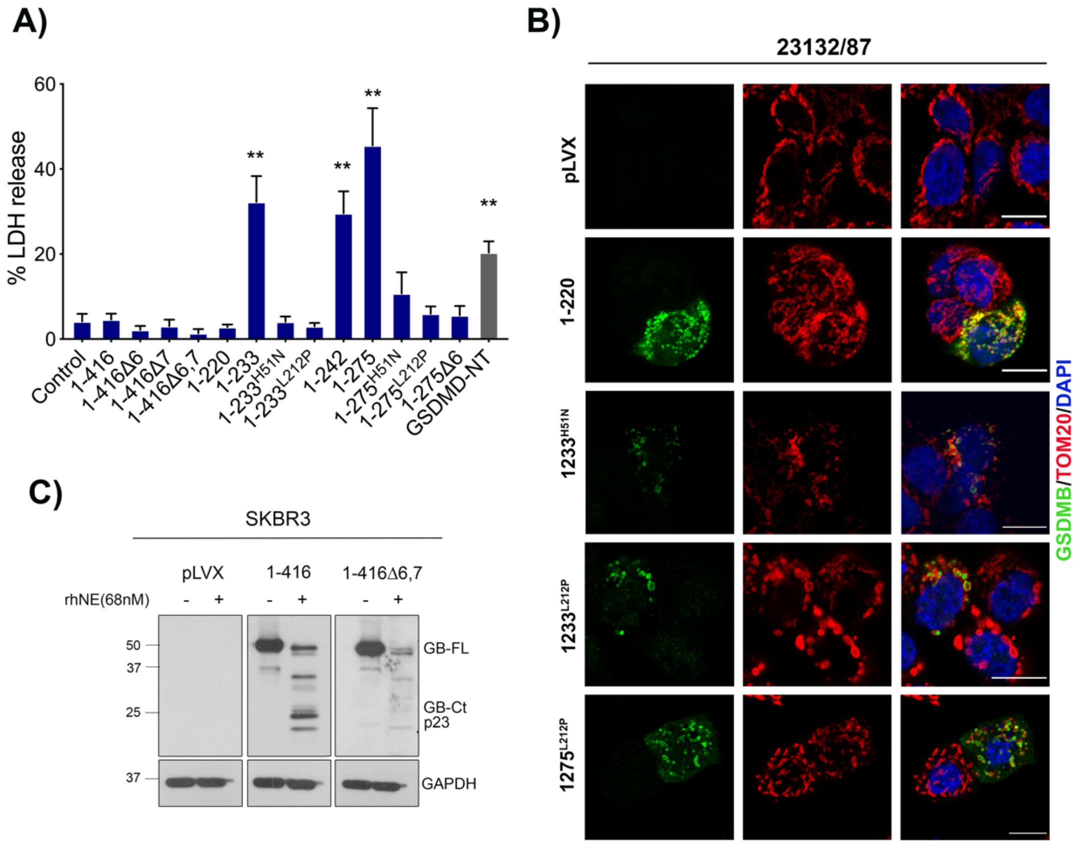

Supplementary Figure 8. Pro-cell death role of GSDMB-NT in cancer cells in an isoform-dependent way. Gastric cancer cell line 23132/87 was transiently transfected with GSDMB constructs during 48h. **A**) Cytotoxicity was measured by lactate dehydrogenase (LDH) assay. Bars represents mean values ± SEM from three independent experiments. Differences between control (empty vector) and each condition was tested by two-tailed unpaired t-test: **p<0,01 and ***p<0,001. GSDMD-NT was used as a positive control. **B**) Immunofluorescence and confocal microscopy analysis in 23132/87 cells transiently transfected with indicated GSDMB-NT constructs. GSDMB-NT (green; NT antibody SIGMA, HPA023925), co-localizes with mitochondrial marker TOM20 in red. pLVX (empty vector) was used as a negative control. **C**) Immunoblotting analysis of GSDMB cleavage by recombinant human neutrophil elastase (rhNE). SKBR3 breast cancer cell lysates containing GSDMB isoforms 2 and 3 (1-275Δ6,7 and 1-275, respectively) were incubated with rhNE (68nM) at 37°C for 30 min. The generated GSDMB-CT p23 fragment was detected using anti-GSDMB-CT antibody Ab-GB (11).

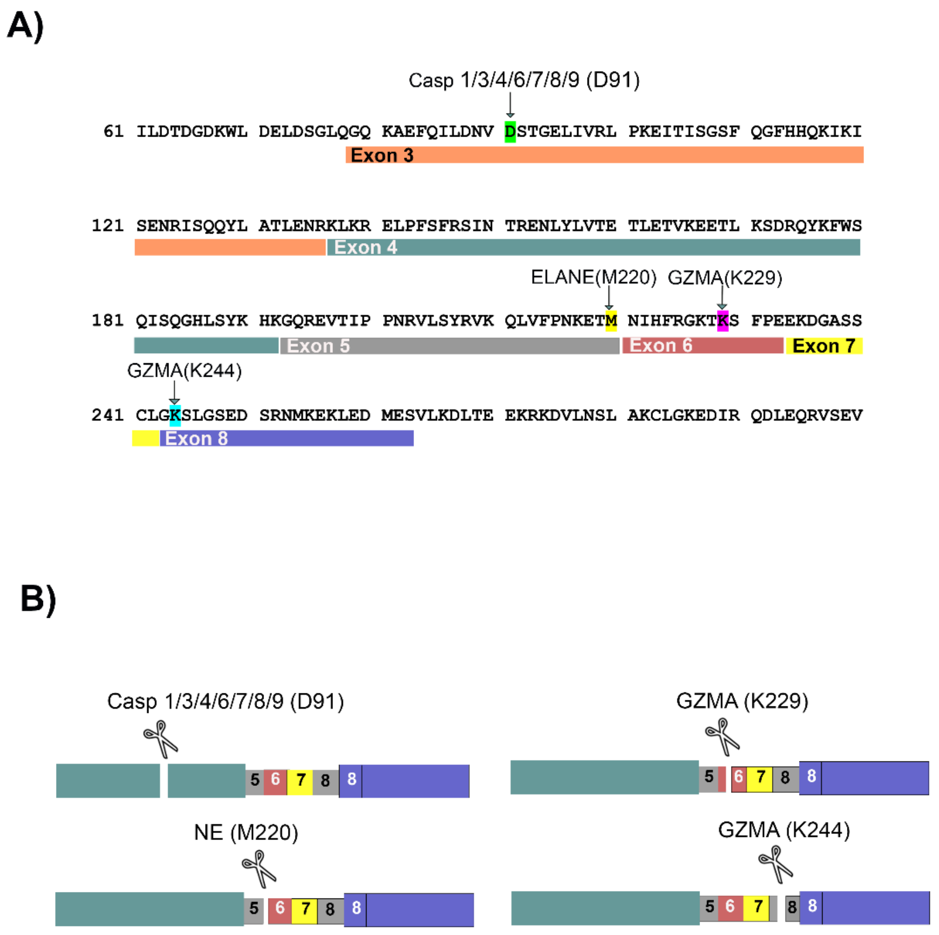

Supplementary Figure 9. GSDMB cleavage by GZMA, Neutrophil Elastase (NE) and caspases. **A**) GSDMB sequence and cleavage sites representation indicating the exon localization. **B**) Schematic representation of GSDMB cleavage by GZMA, NE and caspases.

SUPPLEMENTARY TABLES:

Supplementary Table 1: Cell lines and culture conditions.

Cell line	Medium conditions	Culture conditions
**HEK 293T**	DMEM, 10% fetal bovine serum, 1% L-glutamine, 1% Penicillium/streptomycin	37°C and 5% CO_2
**SKBR3**	RPMI, 10% fetal bovine serum, 1% L-glutamine, 1% Penicillium/streptomycin	37°C and 5% CO_2
**23132/87**	RPMI, 10% fetal bovine serum, 1% L-glutamine, 1% Penicillium/streptomycin	37°C and 5% CO_2
**THP1**	RPMI, 10% fetal bovine serum, 1% L-glutamine 1% Penicillium/streptomycin	37°C and 5% CO_2
**NK-92**	75% Alfa-MEM, 12,5% fetal bovine serum, 12,5% horse serum, 2mM L-glutamine, 5ng/ml IL-2	37°C and 5% CO_2

Supplementary Table 2: Key resources table.

Reagent or resource	Source	Identifier	Probe and dilution
Antibodies			
anti Caspase 3	Santa Cruz Biotech	sc-56053	WB 1:250
anti GAPDH	Calbiochem	CB1001	WB 1:50 000
anti Gasdermin B	in house	Ab-GB (7)	WB 1:250
anti Gasdermin B	Abcam	ab215729	WB 1:1 000
anti Gasdermin B	Santa Cruz Biotech	sc-101239	WB 1:250
anti Gasdermin B	Sigma Aldrich	HPA023925	IF 1:50
anti GFP	Invitrogen	A11122	WB 1:2 000
anti GM130	BD BIOSCIENCES	610822	IF 1:100
anti Granzyme A	Abcam	ab209205	WB 1:1 000
anti HA	Sigma Aldrich	12158167001	WB 1:500
anti Hsp90a/b	Santa Cruz Biotech	sc-13119	WB 1:500
anti human Neutrophile Elastase	Santa Cruz Biotech	sc-55549	WB 1:500
anti LAMP1	Santa Cruz Biotech	sc-20011	IF 1:200
anti myc Tag (mouse)	Cell Signaling	2276	WB 1:1 000
anti myc Tag (rabbit)	Cell Signaling	2278S	WB 1:1 000
anti Trap1/Hsp75	Santa Cruz Biotech	sc-390061	IF 1:100/WB 1:50
anti TOM20	Santa Cruz Biotech	sc-17764	IF 1:200/WB 1:200
anti α1 Sodium Potassium ATPase	Abcam	ab7671	WB 1:10 000
anit α-Tubulin	Sigma Aldrich	T9026	WB 1:10 000
Mouse IgGκ light chain	Santa Cruz Biotech	sc-516102	WB 1:3 000
Mouse anti-rabbit	Santa Cruz Biotech	sc-2357	WB 1:3 000
Goat anti-rat	Nordic Mubio	GARa/IgG2a/PO	WB 1:3 000
Cell lines			
HEK293T	ATCC- American Type Culture Collection	ATCC CRL-3216	
SKBR3	DSMZ-German Collection of Microorganisms	ACC 736	
23132/87	DSMZ-German Collection of Microorganisms	ACC 201	
NK-92	DSMZ-German Collection of Microorganisms	ACC 488	
THP1	ATCC- American Type Culture Collection	ATCC TIB-202	
Plasmids			
pEZ-M61	Genecopoeia	EX-NEG-M61	
pLVX-Puro-MYC	Clontech, Takara	632164	
Lenti-TRE3G-GFP	Transomic	TLO3092-TRE3G-ORF-C-TagGFP2-PGK-Tet3G-puro	
pCS2-3XFlag-hGSDMD	Dr. Pablo Pelegrin	-	
pCMV6-XL4	Origene	NC1720451	
qRT PCR reagents and probes			
GSDMB ISOFORM1	Applied Biosystems™	Hs00938445_m1	
GSDMB ISOFORM2	Applied Biosystems™	Hs00939390_m1	
GSDMB ISOFORM3 and 4	Applied Biosystems™	Hs00940508_m1	
GAPDH	Applied Biosystems™	Hs02758991_g1	
TaqMan® Fast Advanced Master Mix	Applied Biosystems™	4444556	

Supplementary Table 3: Summary table of functional activity of GSDMB constructs and subcellular localization.

GSDMB constructions	Cell localization (HEK293T)	Cytotoxicity (LDH)	Mitochondrial damage (mitoSOX)	Membrane Potential (TMRE)
1-416	Diffuse cytoplasmic	No	No	No
1-416 Δ6	Diffuse cytoplasmic	No	No	No
1-416 Δ7	Diffuse cytoplasmic	No	No	No
1-416 Δ6,7	Diffuse cytoplasmic	No	No	No
$1\text{-}416^{A340D}$	Diffuse cytoplasmic	No	No	ND
92-416	Diffuse cytoplasmic	No	No	ND
1-220	Mitochondrial aggregates	No	No	No
1-233	No detected	High*	High*	Decreased*
$1\text{-}233^{H51N}$	Mitochondrial aggregates	No	No	No
$1\text{-}233^{L212P}$	Mitochondrial aggregates	No	No	No
1-242	No detected	High*	High*	Decreased*
1-275	No detected	High*	High*	Decreased*
$1\text{-}275^{H51N}$	Mitochondrial aggregates	No	No	No
$1\text{-}275^{L212P}$	Mitochondrial aggregates	Moderate*	No	No
$1\text{-}275^{K229A}$	No detected	High*	High*	ND
$1\text{-}275^{K244A}$	No detected	High*	High*	ND
1-275, Δ6	Diffuse cytoplasmic	No	No	No
1-275 Δ7	No detected	High*	High*	No
1-275 Δ6,7	Diffuse cytoplasmic	No	No	No
$1\text{-}275^{4AE6}$	Diffuse cytoplasmic	No	No	ND

* p<0,05; No = no significant effect compared to control conditions. ND = not done.

SUPPLEMENTARY VIDEOS LEGENDS:

Supplementary Video 1. Live-cell imaging of HEK293T cells expressing 1-416-GFP after 2 hours of pre-induction with doxycycline. Cells were recorded for at least 20h (1 picture every 10 min). Propidium Iodide (0.2µg/ml in red) stains nuclei from dying cells. Bright field shown. Scale bar represents 10µm.

Supplementary Video 2. Live-cell imaging of HEK293T cells expressing 1-416-GFP after 2 hours of pre-induction with doxycycline. Cells were recorded for at least 20h (1 picture every 10 min). Propidium Iodide (0.2µg/ml in red) stains nuclei from dying cells. Scale bar represents 10µm.

Supplementary Video 3. Live-cell imaging of HEK293T cells expressing 1-220-GFP after 2 hours of pre-induction with doxycycline. Cells were recorded for at least 20h (1 picture every 10 min). Propidium Iodide (0.2µg/ml in red) stains nuclei from dying cells. Bright field shown. Scale bar represents 10µm.

Supplementary Video 4. Live-cell imaging of HEK293T cells expressing 1-220-GFP after 2 hours of pre-induction with doxycycline. Cells were recorded for at least 20h (1 picture every 10 min). Propidium Iodide (0.2µg/ml in red) stains nuclei from dying cells. Scale bar represents 10µm.

Supplementary Video 5. Live-cell imaging of HEK293T cells expressing 1-242Δ6-GFP after 2 hours of pre-induction with doxycycline. Cells were recorded for at least 20h (1 picture every 10 min). Propidium Iodide (0.2µg/ml in red) stains nuclei from dying cells. Bright field shown. Scale bar represents 10µm.

Supplementary Video 6. Live-cell imaging of HEK293T cells expressing 1-242Δ6-GFP after 2 hours of pre-induction with doxycycline. Cells were recorded for at least 20h (1 picture every 10 min). Propidium Iodide (0.2µg/ml in red) stains nuclei from dying cells. Scale bar represents 10µm.

Supplementary Video 7. Live-cell imaging of HEK293T cells expressing 1-275Δ6-GFP after 2 hours of pre-induction with doxycycline. Cells were recorded for at least 20h (1 picture every 10 min). Propidium Iodide (0.2µg/ml in red) stains nuclei from dying cells. Bright field shown. Scale bar represents 10µm.

Supplementary Video 8. Live-cell imaging of HEK293T cells expressing 1-275Δ6-GFP after 2 hours of pre-induction with doxycycline. Cells were recorded for at least 20h (1 picture every 10 min). Propidium Iodide (0.2µg/ml in red) stains nuclei from dying cells. Scale bar represents 10µm.

Supplementary Video 9. Live-cell confocal imaging over 1-2 hours of HEK293T cells expressing 1-242-GFP following doxycycline induction. Propidium Iodide (0.2µg/ml in red) stains nuclei from dying cells. Bright field shown. Scale bar represents 10µm.

Supplementary Video 10. Live-cell confocal imaging over 1-2 hours of HEK293T cells expressing 1-242-GFP following doxycycline induction. Propidium Iodide (0.2µg/ml in red) stains nuclei from dying cells. Scale bar represents 10µm.

Supplementary Video 11. Live-cell confocal imaging over 1-2 hours of HEK293T cells expressing 1-275-GFP following doxycycline induction. Propidium Iodide (0.2µg/ml in red) stains nuclei from dying cells. Bright field shown. Scale bar represents 10µm.

Supplementary Video 12. Live-cell confocal imaging over 1-2 hours of HEK293T cells expressing 1-275-GFP following doxycycline induction. Propidium Iodide (0.2µg/ml in red) stains nuclei from dying cells. Scale bar represents 10µm.

UNCROPPED WESTERN BLOTS:

Figure 3B

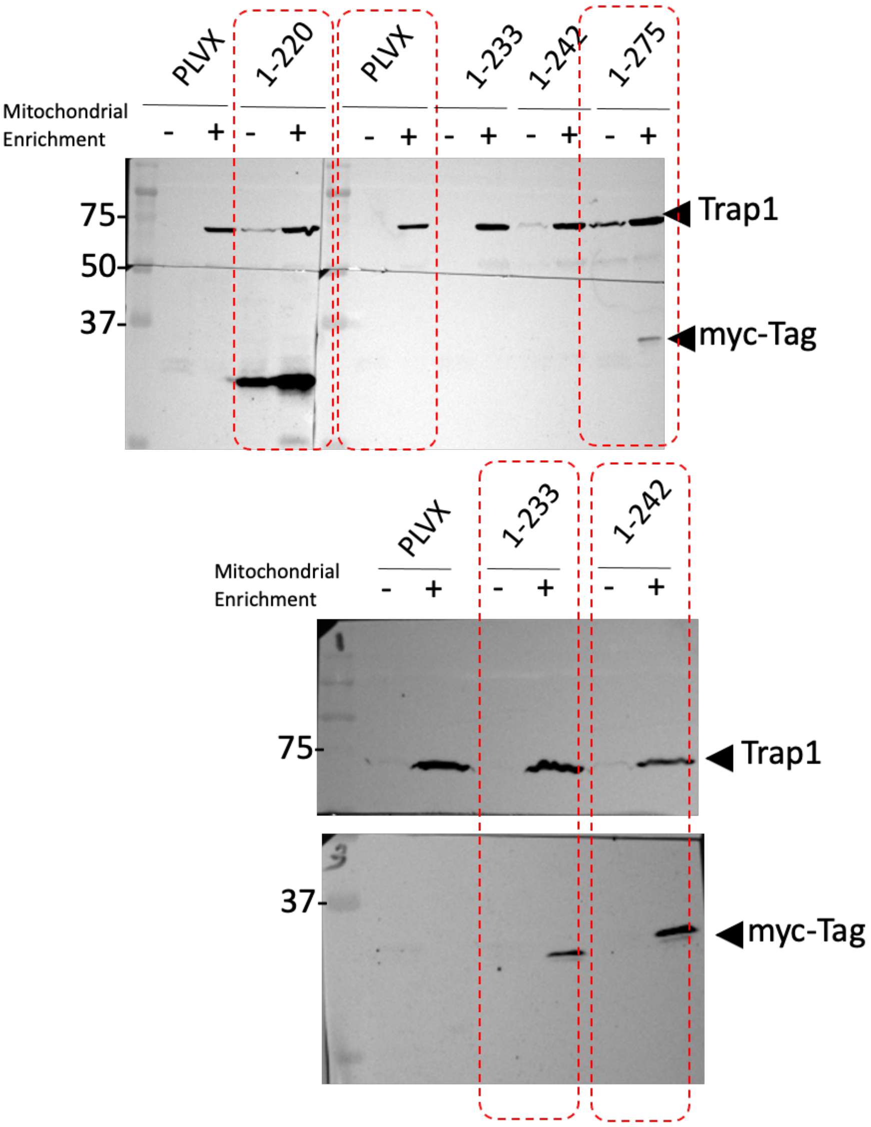

Figure 4D

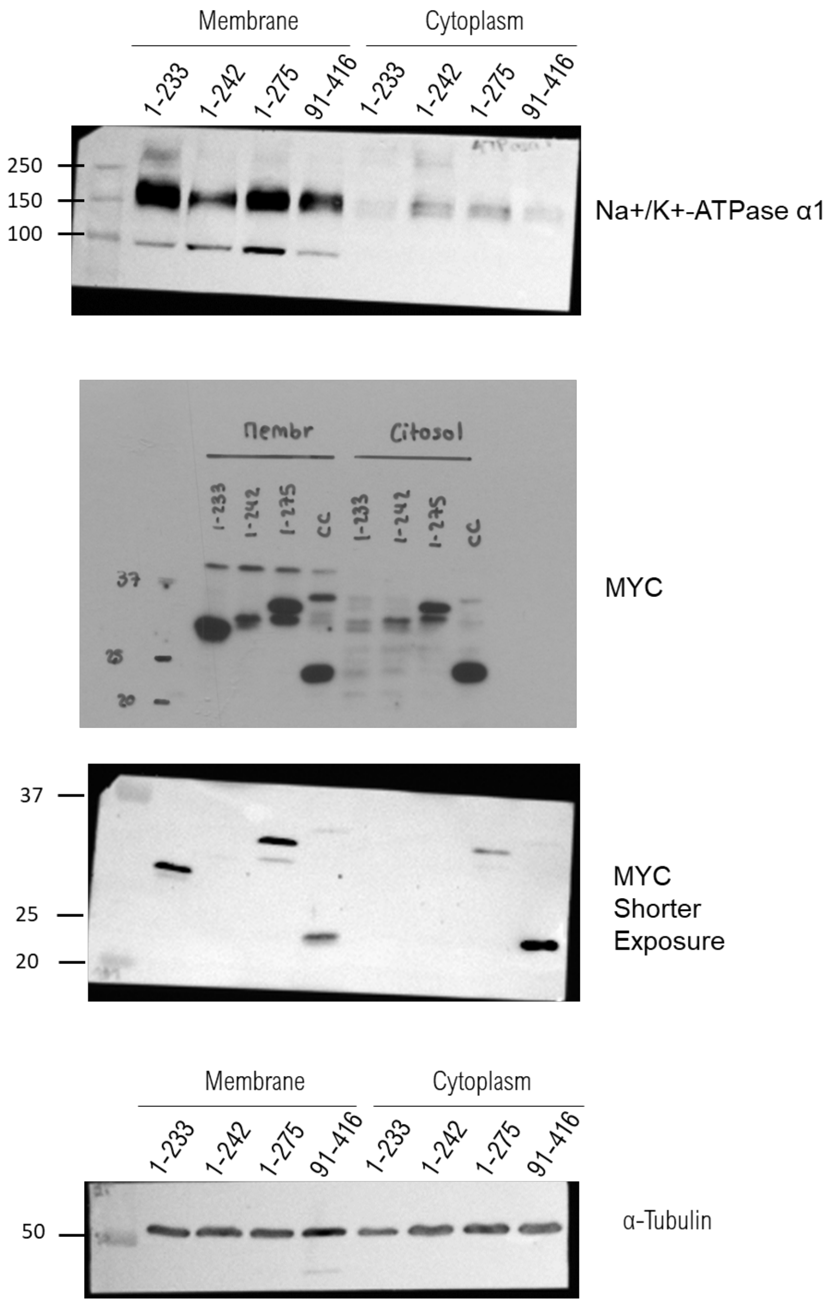
Membrane
Cytoplasm
1-233
1-242
1-275
91-416
1-233
1-242
1-275
91-416
250
150
100
Na+/K+-ATPase α1
Membr
Citosol
1-233
1-242
1-275
cc
1-233
1-242
1-275
cc
37
25
20
MYC
37
25
20
MYC
Shorter
Exposure
Membrane
Cytoplasm
1-233
1-242
1-275
91-416
1-233
1-242
1-275
91-416
50
α-Tubulin

Figure 5B

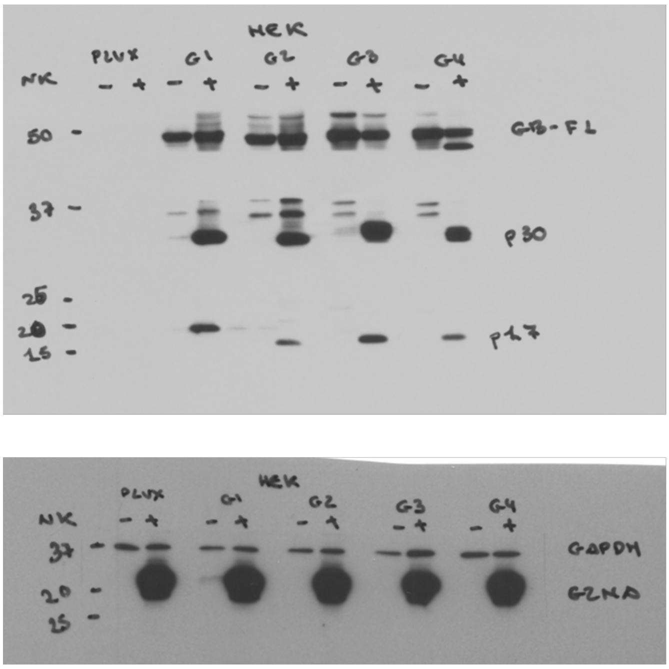

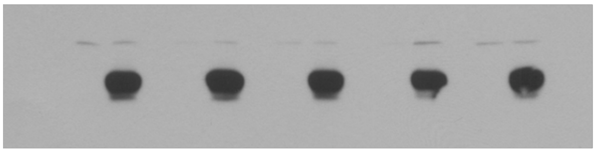

GZMA
Shorter Exposure

Figure 5E

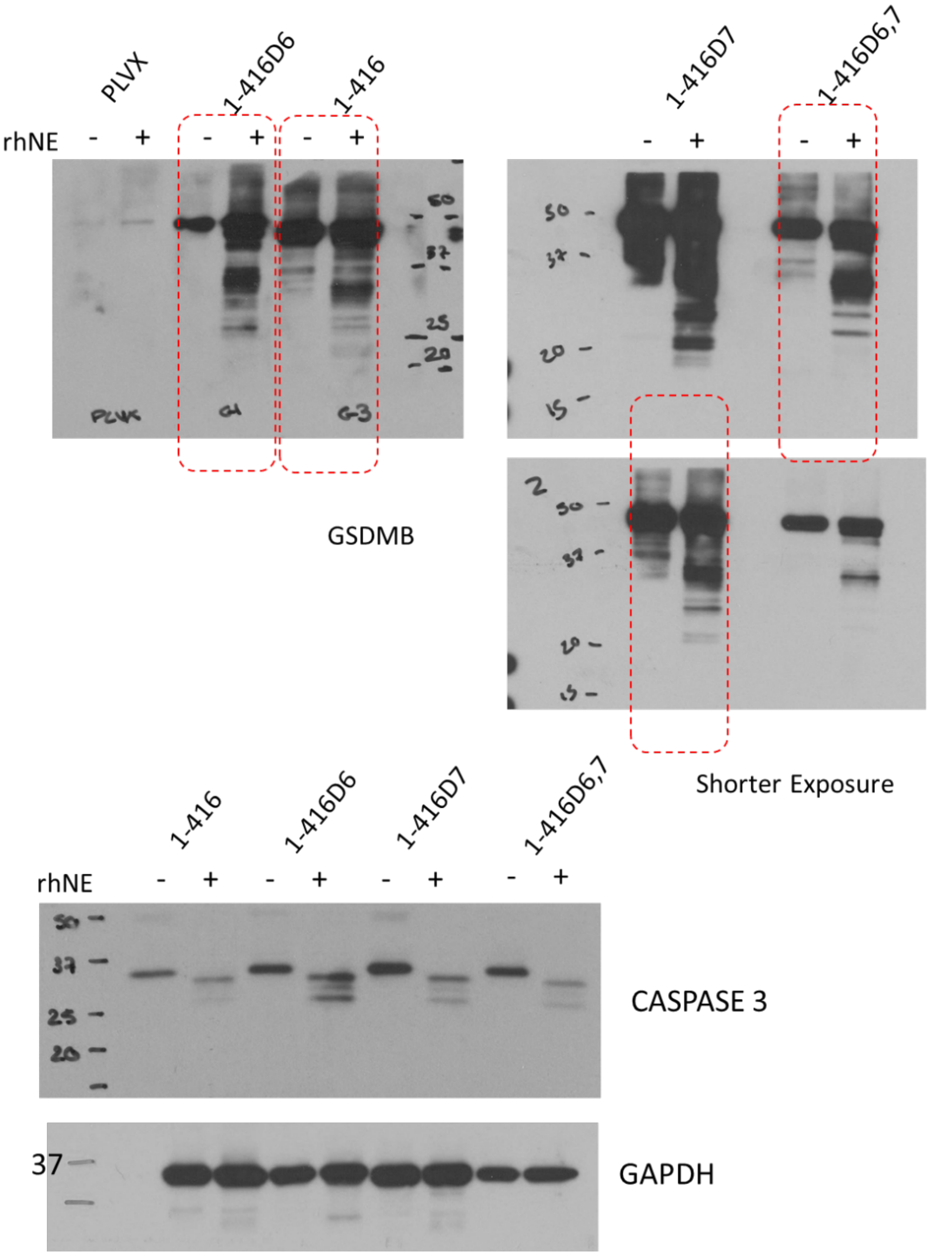
PLVX
1-416D6
1-416
1-416D7
1-416D6,7
rhNE
- + - + - +
- + - +
50
37
25
20
15
GSDMB
Shorter Exposure
1-416
1-416D6
1-416D7
1-416D6,7
rhNE
- + - + - + - +
50
37
25
20
CASPASE 3
37
GAPDH

Figure 5F

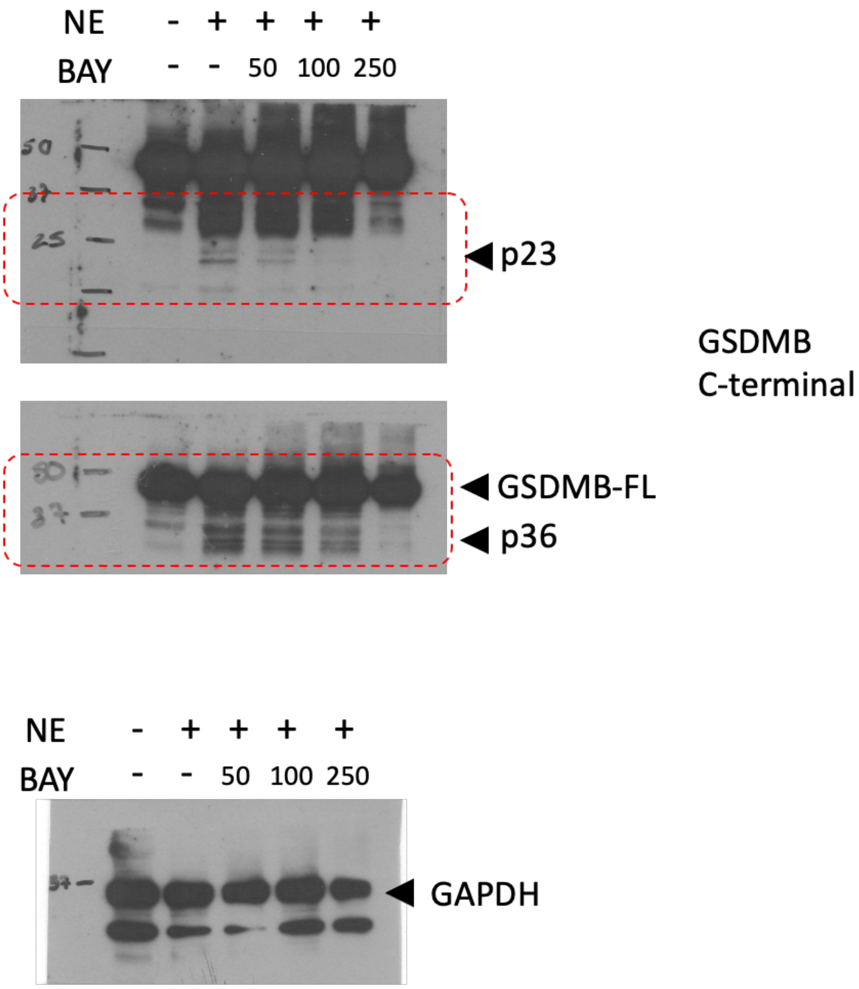
NE - + + + +
BAY - - 50 100 250
p23
GSDMB-FL
p36
GSDMB
C-terminal
NE - + + + +
BAY - - 50 100 250
GAPDH

Figure 5H

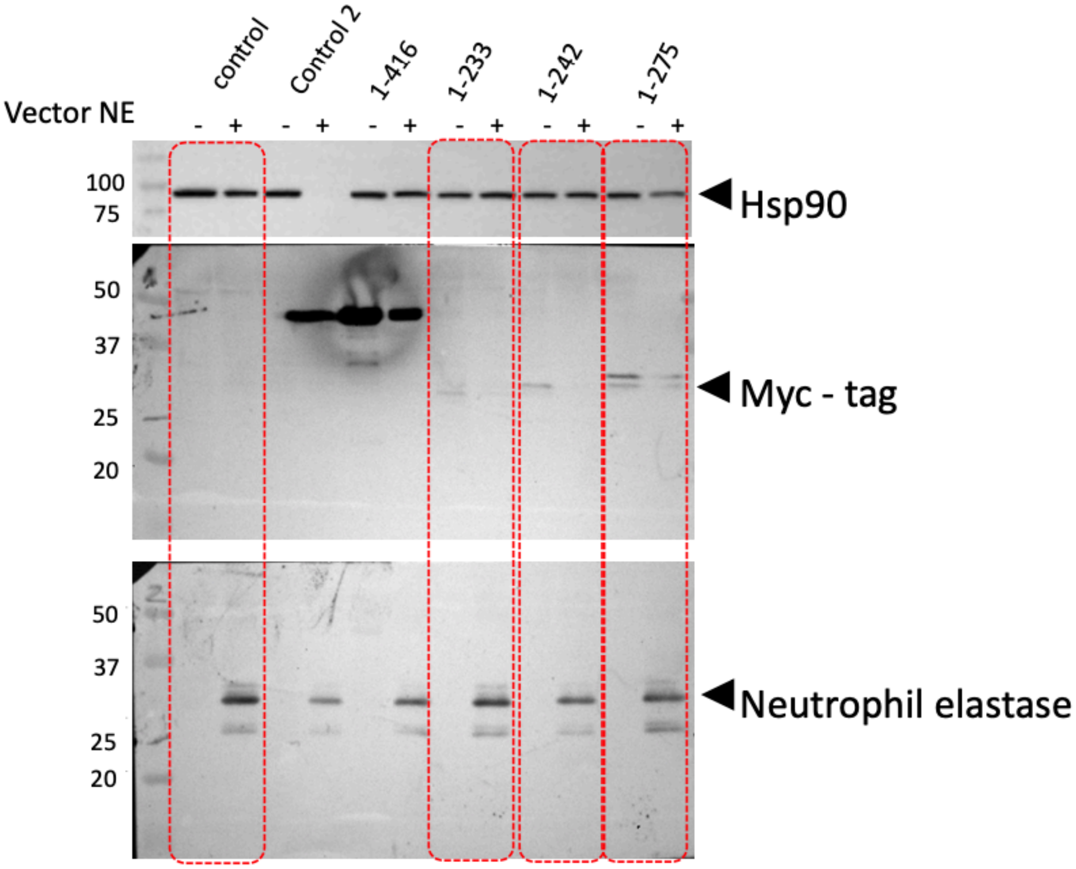

control
Control 2
1-416
1-233
1-242
1-275
Vector NE
- + - + - + - + - + - +
100
75
Hsp90
50
37
25
20
Myc - tag
50
37
25
20
Neutrophil elastase

Figure 6D

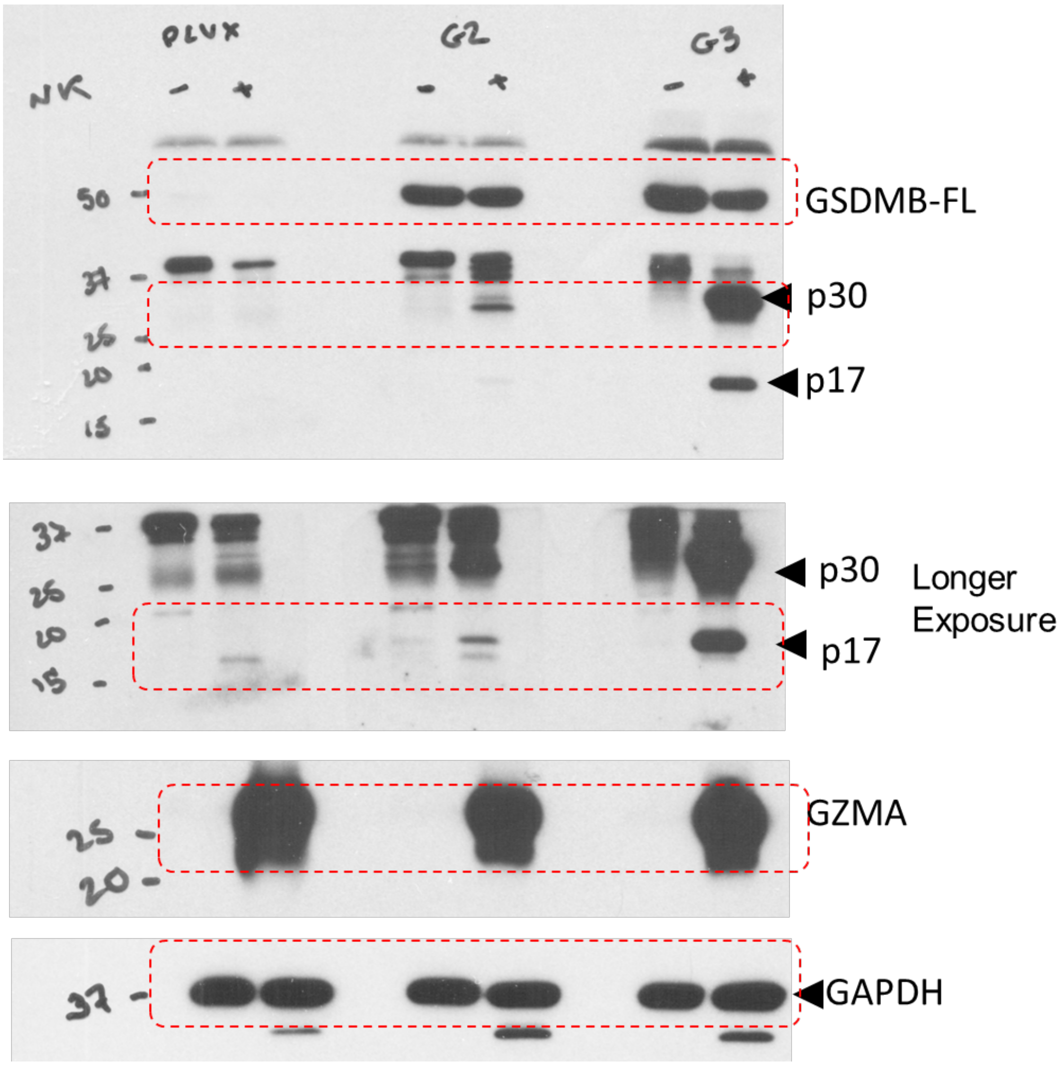

pLUX
G2
G3
NK
-
+
50
37
25
20
15
GSDMB-FL
p30
p17
Longer Exposure
GZMA
GAPDH

Supplementary Figure 1

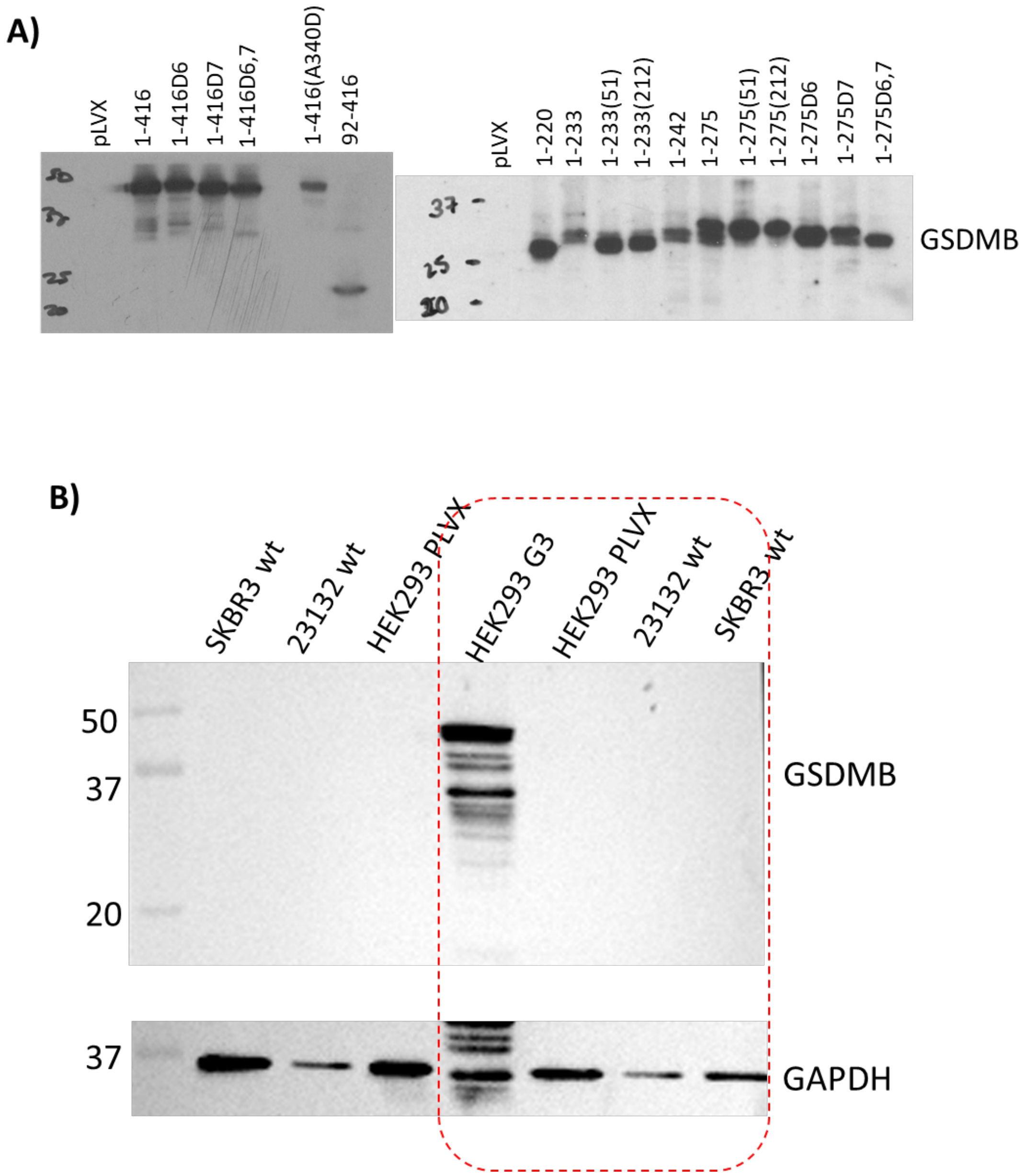

Supplementary Figure 6

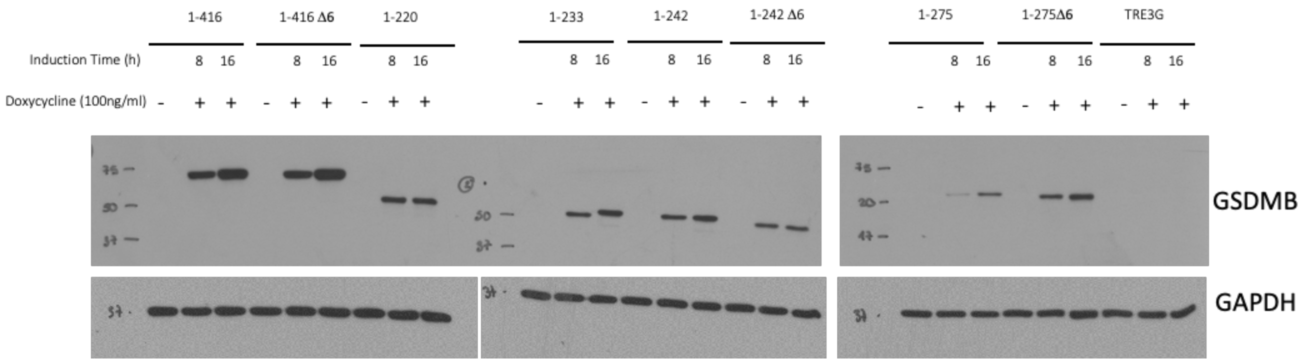

Supplementary Figure 7

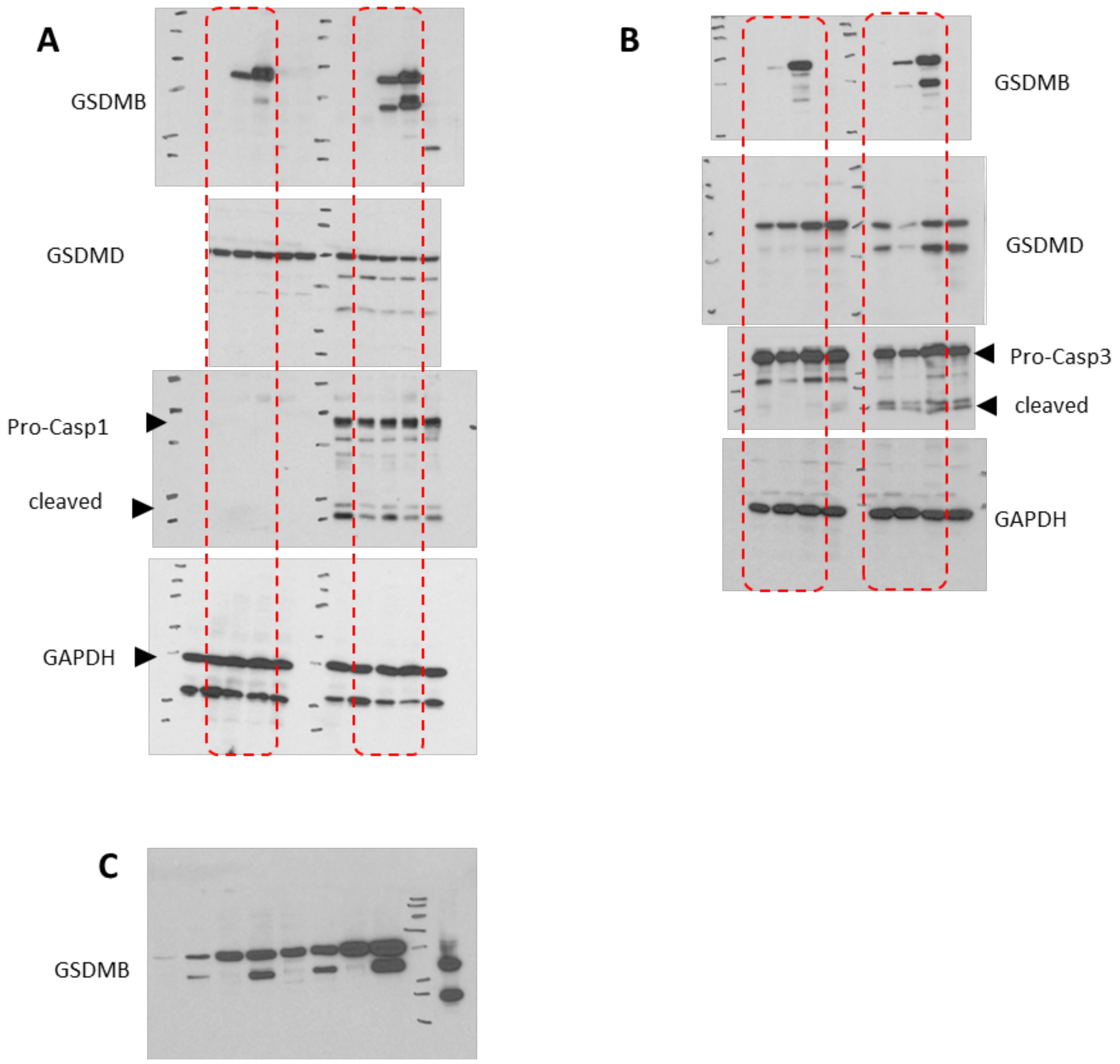

Supplementary Figure 8

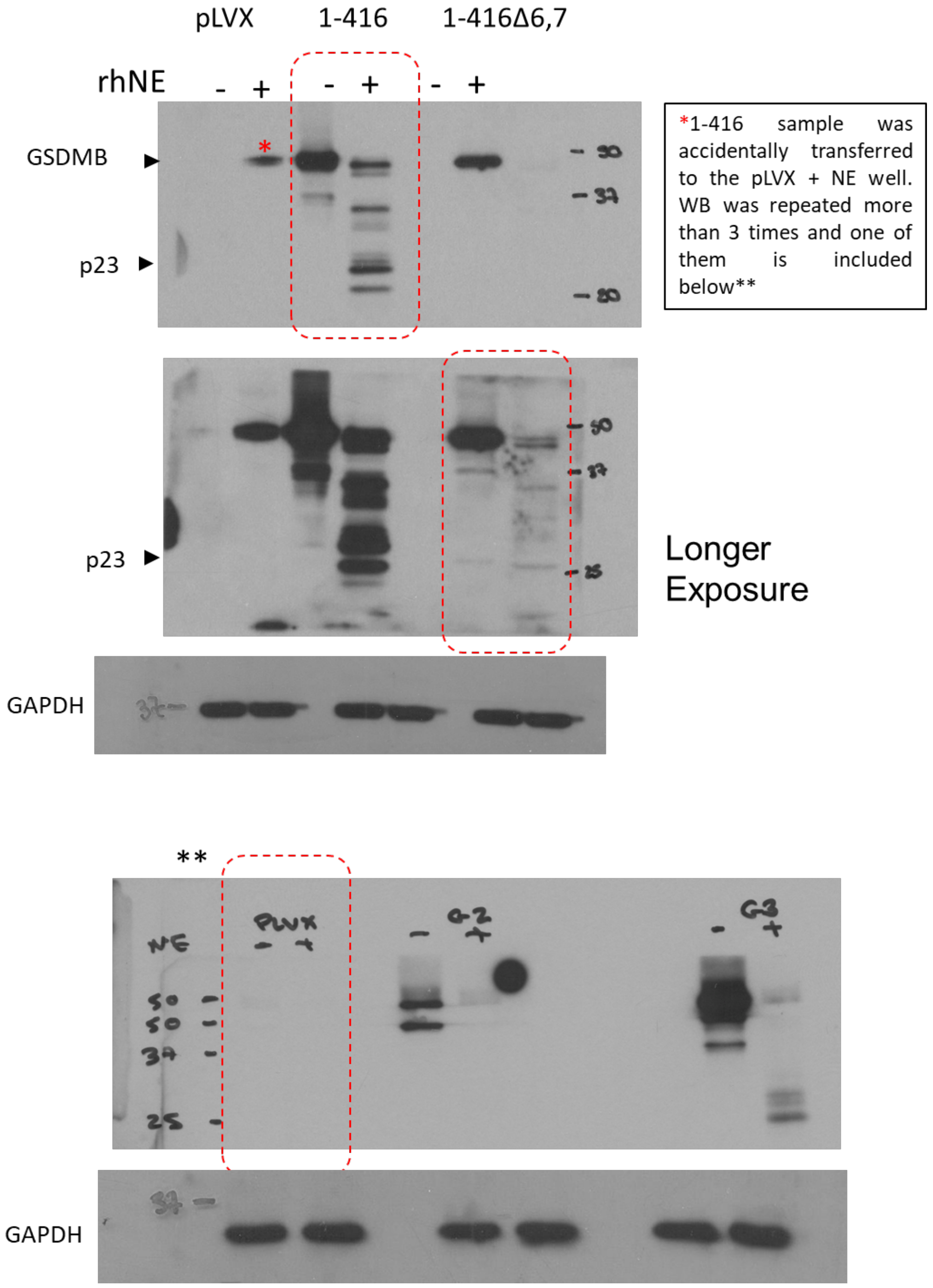

REFERENCES CITED IN THE SUPPLEMENTARY INFORMATION

1. Ding J, Wang K, Liu W, She Y, Sun Q, Shi J, et al. Pore-forming activity and structural autoinhibition of the gasdermin family. Nature. 2016;535(7610):111-6.
2. Fernández-Vizarra E, Ferrín G, Pérez-Martos A, Fernández-Silva P, Zeviani M, Enríquez JA. Isolation of mitochondria for biogenetical studies: An update. Mitochondrion. 2010;10(3):253-62.
3. Bronner DN, O'Riordan MX. Measurement of Mitochondrial DNA Release in Response to ER Stress. Bio Protoc. 2016;6(12).
4. Waterhouse A, Bertoni M, Bienert S, Studer G, Tauriello G, Gumienny R, et al. SWISS-MODEL: homology modelling of protein structures and complexes. Nucleic Acids Res. 2018;46(W1):W296-W303.
5. Ruan J, Xia S, Liu X, Lieberman J, Wu H. Cryo-EM structure of the gasdermin A3 membrane pore. Nature. 2018;557(7703):62-7.
6. Xia S, Zhang Z, Magupalli VG, Pablo JL, Dong Y, Vora SM, et al. Gasdermin D pore structure reveals preferential release of mature interleukin-1. Nature. 2021;593(7860):607-11.
7. Marcos-Alcalde Í, Mendieta-Moreno JI, Puisac B, Gil-Rodríguez MC, Hernández-Marcos M, Soler-Polo D, et al. Two-step ATP-driven opening of cohesin head. Sci Rep. 2017;7(1):3266.
8. Shevchenko A, Wilm M, Vorm O, Mann M. Mass spectrometric sequencing of proteins silver-stained polyacrylamide gels. Anal Chem. 1996;68(5):850-8.
9. Pérez M, García-Limones C, Zapico I, Marina A, Schmitz ML, Muñoz E, et al. Mutual regulation between SIAH2 and DYRK2 controls hypoxic and genotoxic signaling pathways. J Mol Cell Biol. 2012;4(5):316-30.
10. Alonso R, Pisa D, Marina AI, Morato E, Rábano A, Rodal I, et al. Evidence for fungal infection in cerebrospinal fluid and brain tissue from patients with amyotrophic lateral sclerosis. Int J Biol Sci. 2015;11(5):546-58.
11. Hergueta-Redondo M, Sarrio D, Molina-Crespo Á, Vicario R, Bernadó-Morales C, Martínez L, et al. Gasdermin B expression predicts poor clinical outcome in HER2-positive breast cancer. Oncotarget. 2016;7(35):56295-308.
12. Taabazuing CY, Okondo MC, Bachovchin DA. Pyroptosis and Apoptosis Pathways Engage in Bidirectional Crosstalk in Monocytes and Macrophages. Cell Chem Biol. 2017;24(4):507-14.e4.
13. Chao KL, Kulakova L, Herzberg O. Gene polymorphism linked to increased asthma and IBD risk alters gasdermin-B structure, a sulfatide and phosphoinositide binding protein. Proc Natl Acad Sci U S A. 2017;114(7):E1128-E37.
14. Chen Q, Shi P, Wang Y, Zou D, Wu X, Wang D, et al. GSDMB promotes non-canonical pyroptosis by enhancing caspase-4 activity. J Mol Cell Biol. 2019;11(6):496-508.
