# Supplementary material for: Distinct GSDMB protein isoforms and protease cleavage processes differentially control pyroptotic cell death and mitochondrial damage in cancer cells
Source: Cell Death Differ. 2023 Mar 11;30(5):1366–81. doi: 10.1038/s41418-023-01143-y (PMC10154425; doi:10.1038/s41418-023-01143-y)
Supplement: Supplementary file 2 — Supplementary videos 1-12 [file 41418_2023_1143_MOESM2_ESM.pptx]

## Slide 1
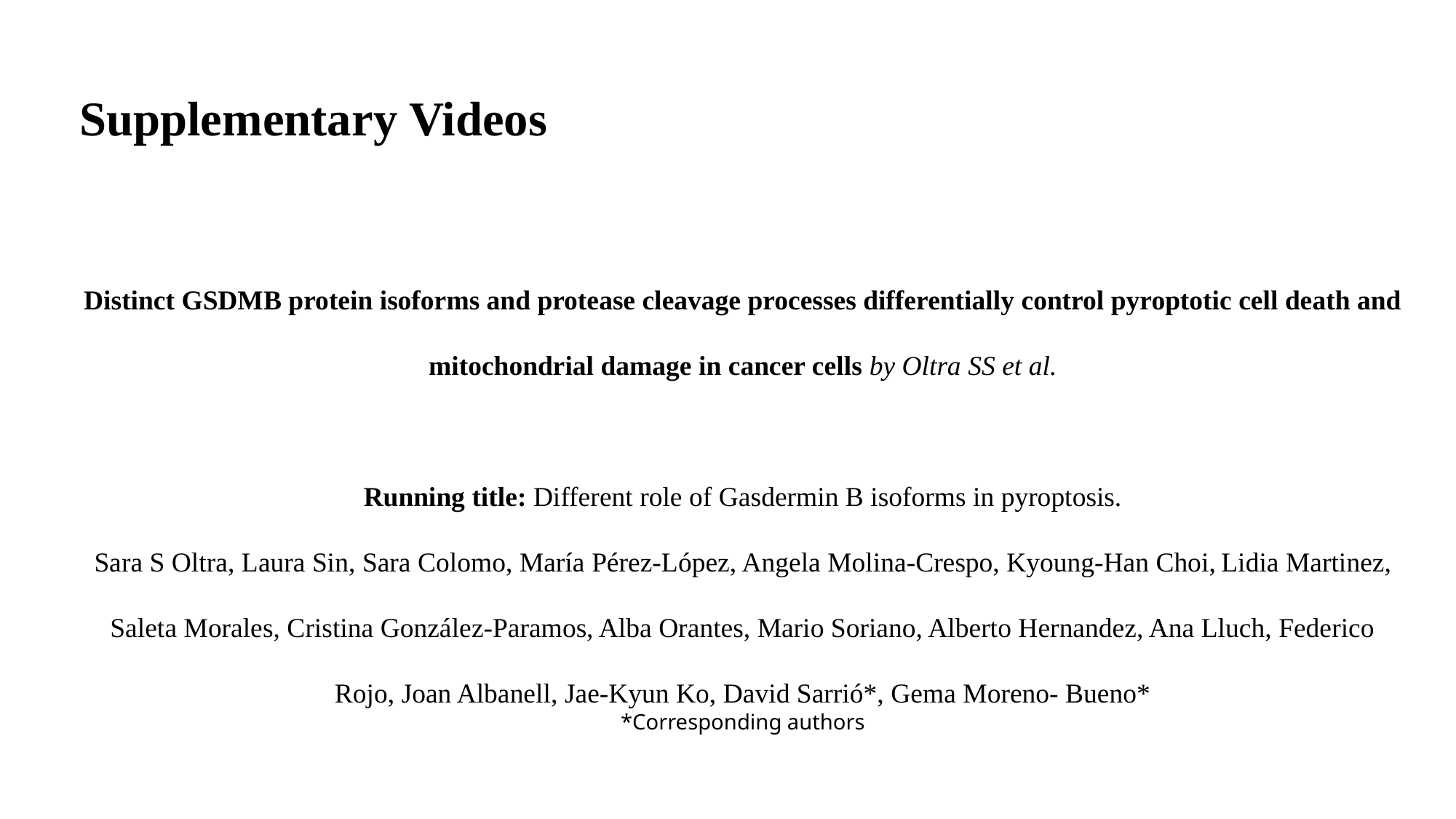

Supplementary Videos
Distinct GSDMB protein isoforms and protease cleavage processes differentially control pyroptotic cell death and mitochondrial damage in cancer cells by Oltra SS et al.
Running title: Different role of Gasdermin B isoforms in pyroptosis.
Sara S Oltra, Laura Sin, Sara Colomo, María Pérez-López, Angela Molina-Crespo, Kyoung-Han Choi, Lidia Martinez, Saleta Morales, Cristina González-Paramos, Alba Orantes, Mario Soriano, Alberto Hernandez, Ana Lluch, Federico Rojo, Joan Albanell, Jae-Kyun Ko, David Sarrió*, Gema Moreno- Bueno*
*Corresponding authors

## Slide 2
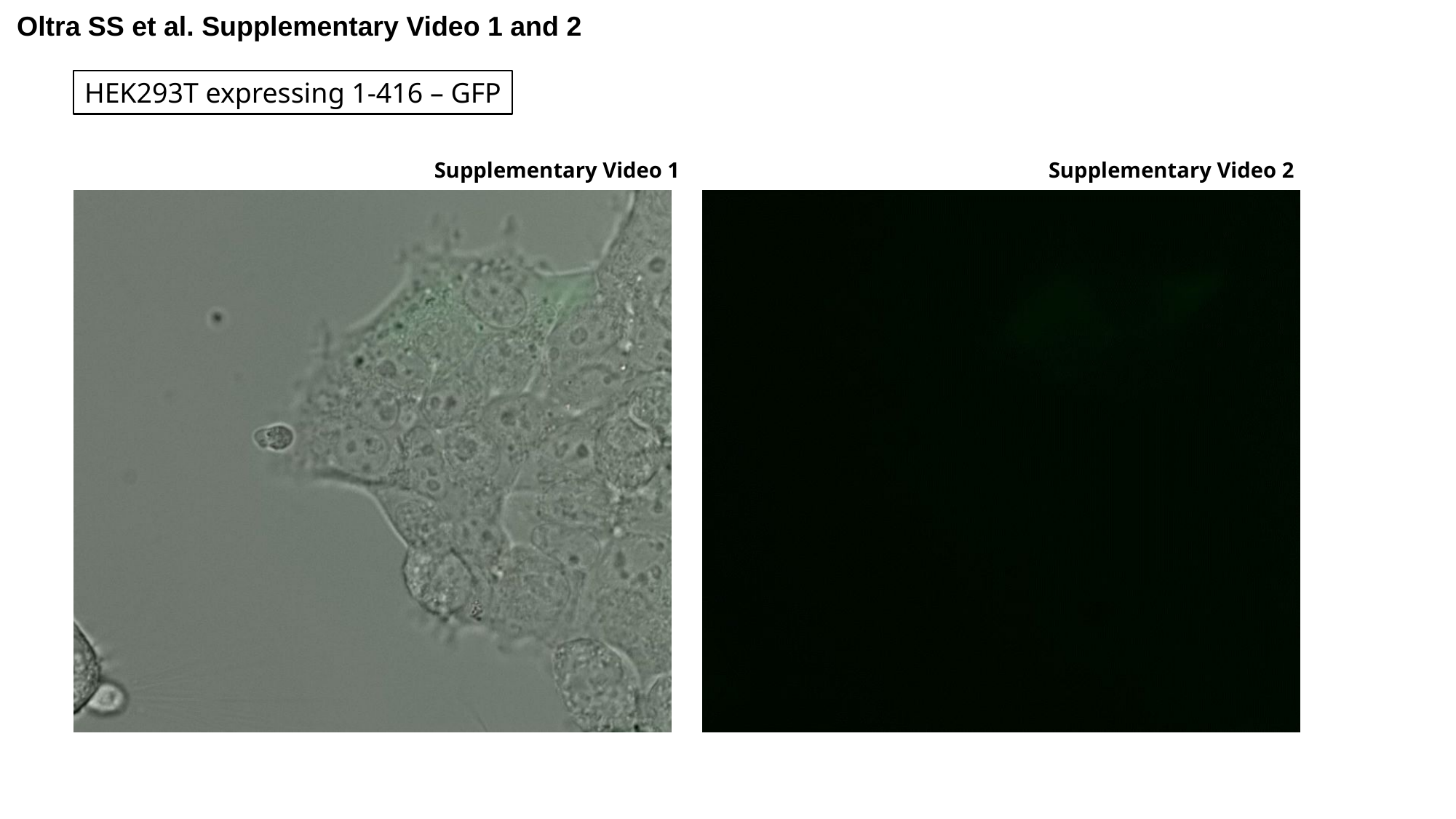

Oltra SS et al. Supplementary Video 1 and 2
HEK293T expressing 1-416 – GFP
Supplementary Video 2
Supplementary Video 1

## Slide 3
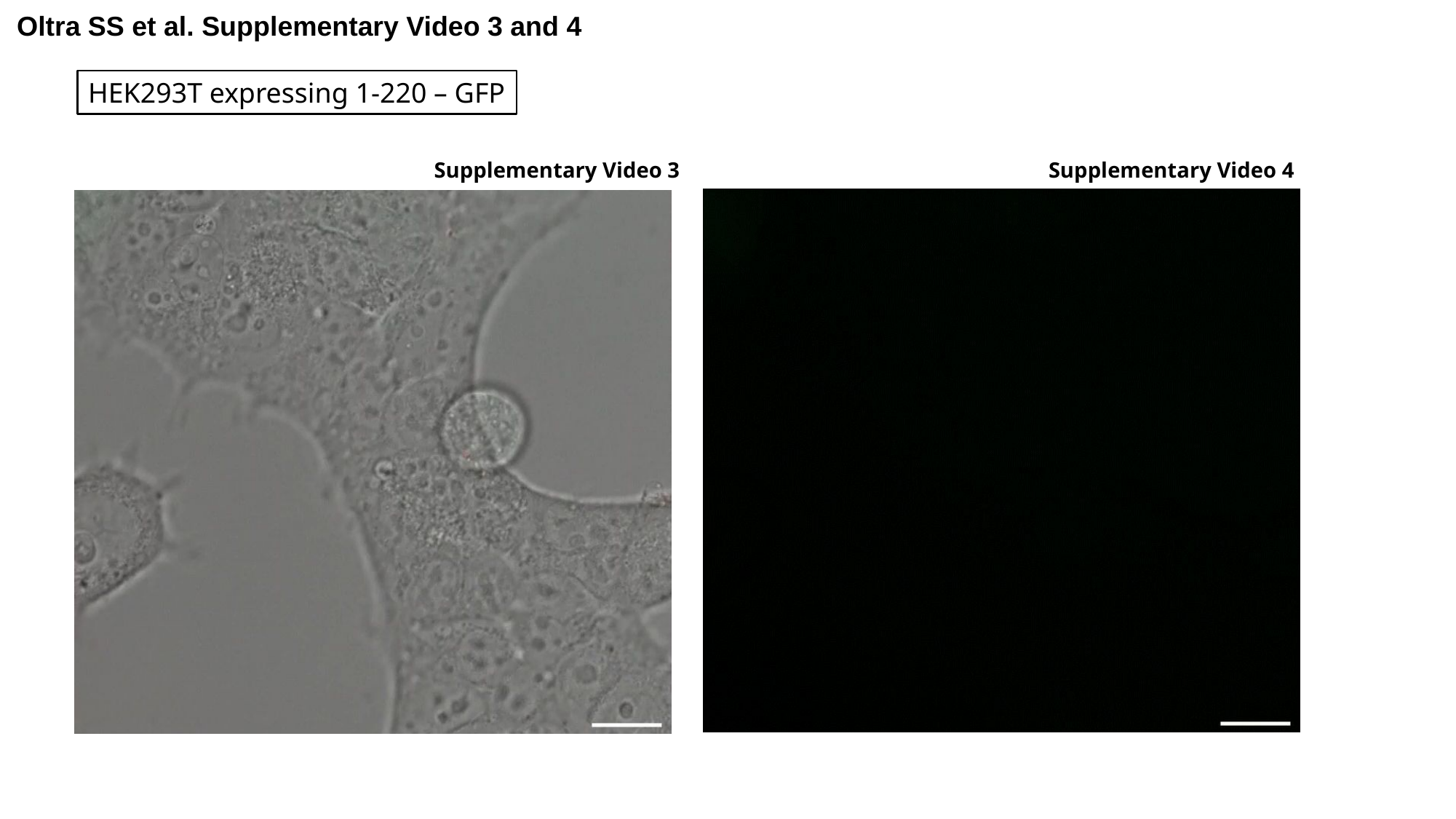

Oltra SS et al. Supplementary Video 3 and 4
HEK293T expressing 1-220 – GFP
Supplementary Video 4
Supplementary Video 3

## Slide 4
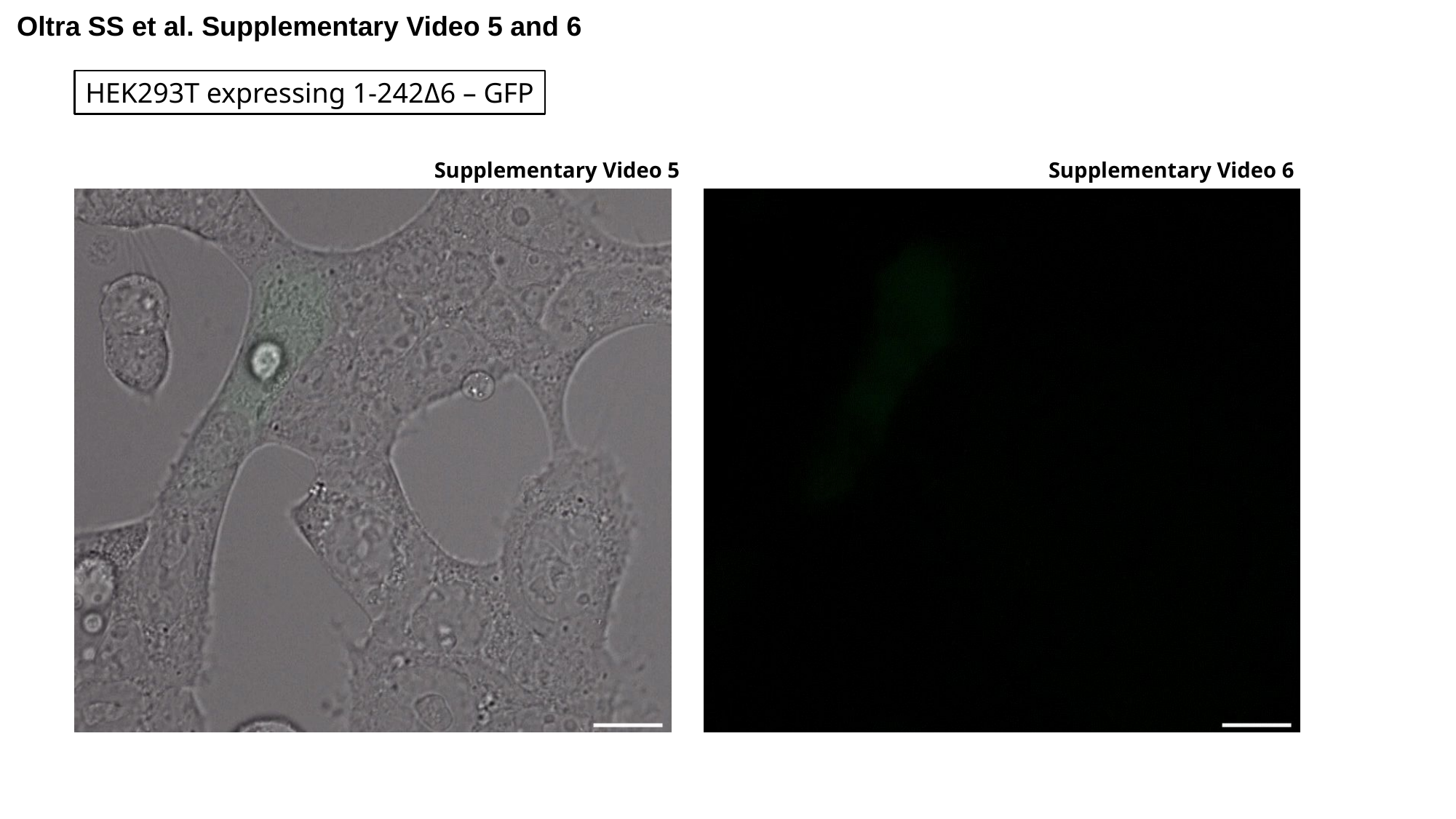

Oltra SS et al. Supplementary Video 5 and 6
HEK293T expressing 1-242Δ6 – GFP
Supplementary Video 6
Supplementary Video 5

## Slide 5
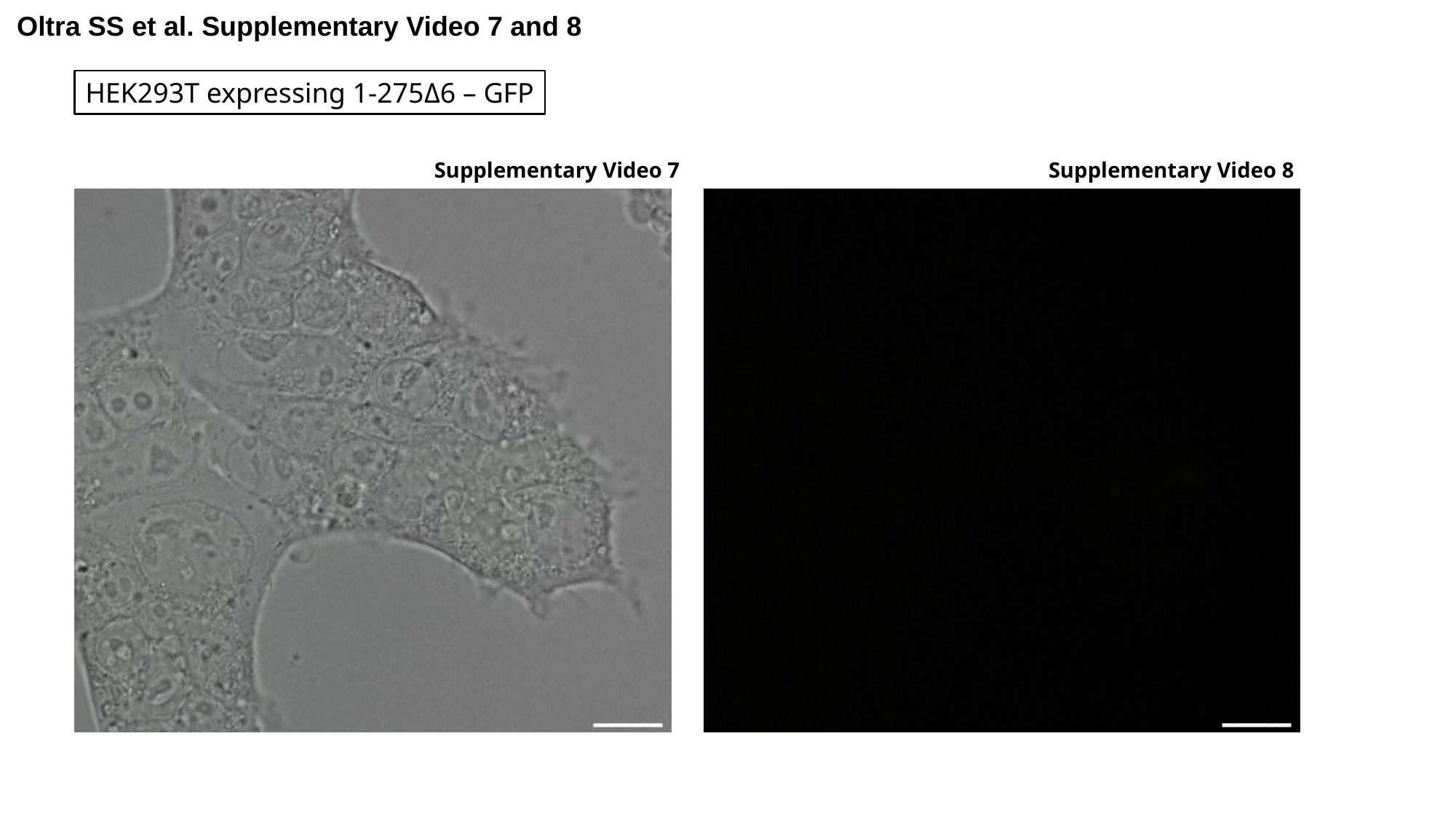

Oltra SS et al. Supplementary Video 7 and 8
HEK293T expressing 1-275Δ6 – GFP
Supplementary Video 8
Supplementary Video 7

## Slide 6
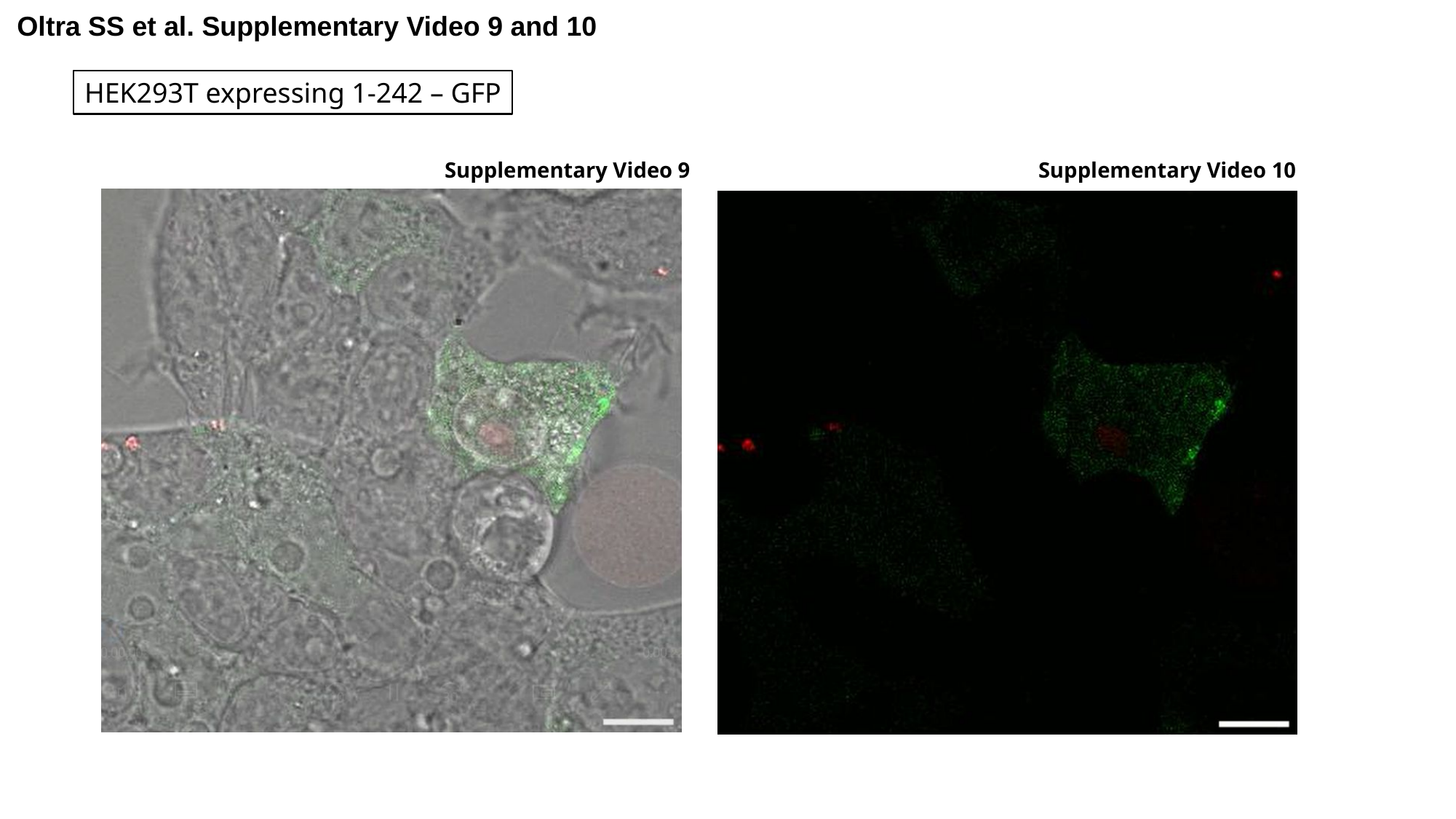

Oltra SS et al. Supplementary Video 9 and 10
HEK293T expressing 1-242 – GFP
Supplementary Video 10
Supplementary Video 9

## Slide 7
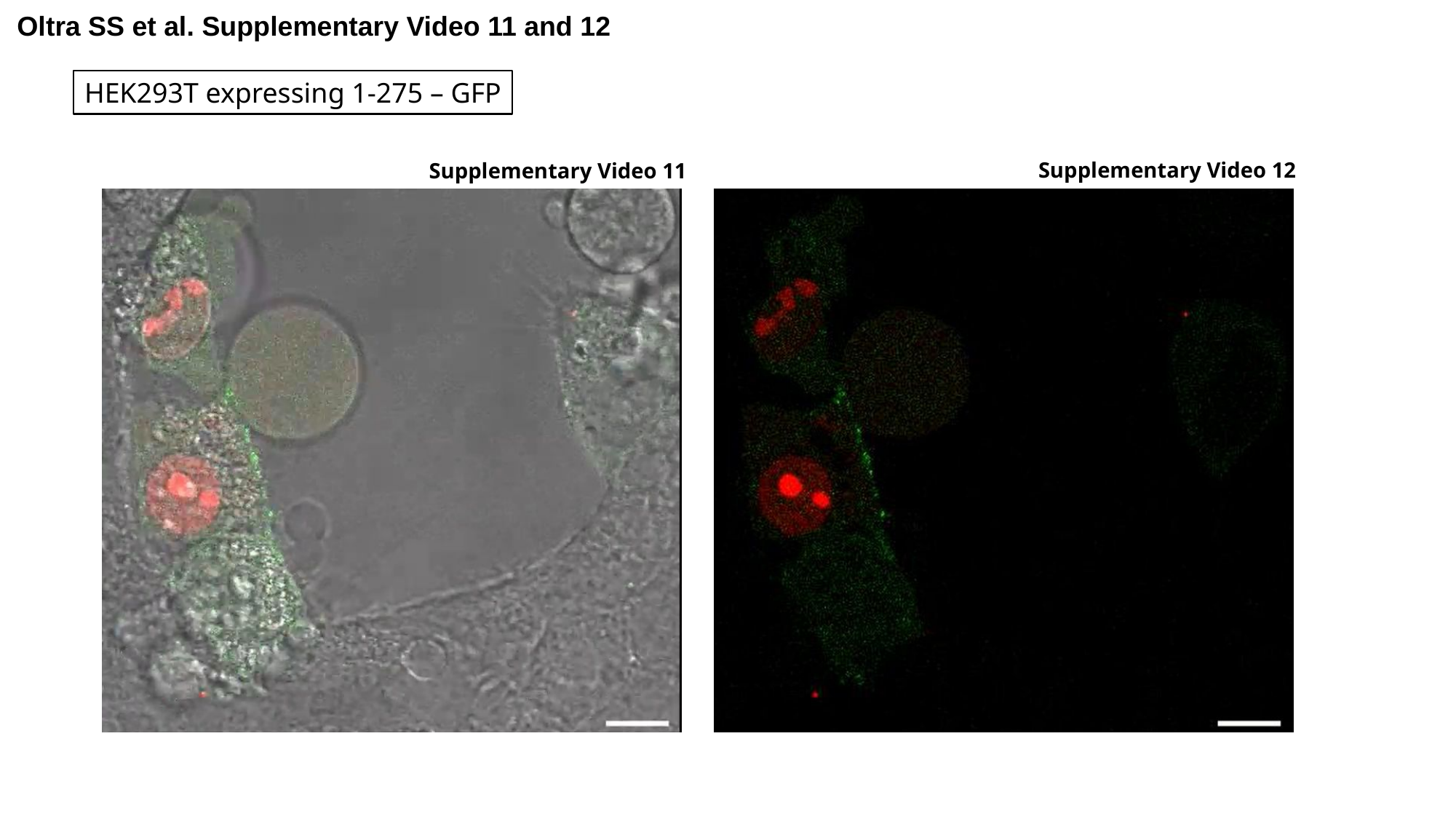

Oltra SS et al. Supplementary Video 11 and 12
HEK293T expressing 1-275 – GFP
Supplementary Video 12
Supplementary Video 11
